# Tuning antiviral CD8 T-cell response via proline-altered peptide ligand vaccination

**DOI:** 10.1371/journal.ppat.1008244

**Published:** 2020-05-04

**Authors:** Adil Doganay Duru, Renhua Sun, Eva B. Allerbring, Jesseka Chadderton, Nadir Kadri, Xiao Han, Kaliroi Peqini, Hannes Uchtenhagen, Chaithanya Madhurantakam, Sara Pellegrino, Tatyana Sandalova, Per-Åke Nygren, Stephen J. Turner, Adnane Achour

**Affiliations:** 1 Science for Life Laboratory, Department of Medicine Solna, Karolinska Institute, and Division of Infectious Diseases, Karolinska University Hospital, Solna, Stockholm, Sweden; 2 NSU Cell Therapy Institute & Dr. Kiran C. Patel College of Allopathic Medicine, Nova Southeastern University, Fort Lauderdale, Florida, United States of America; 3 Department of Microbiology, Biomedical Discovery Institute, Monash University, Clayton, Australia; 4 DISFARM, Dipartimento di Scienze Farmaceutiche, Sezinone Chimica Generale e Organica, Università degli Studi, Milano, Italy; 5 Structural and Molecular Biology Laboratory, Department of Biotechnology, TERI, School of Advanced Studies, New Delhi, India; 6 Division of Protein Engineering, Department of Protein Science, School of Engineering Sciences in Chemistry, Biotechnology and Health, AlbaNova University Center, Royal Institute of Technology, Stockholm, Sweden; Vaccine Research Center, UNITED STATES

## Abstract

Viral escape from CD8^+^ cytotoxic T lymphocyte responses correlates with disease progression and represents a significant challenge for vaccination. Here, we demonstrate that CD8^+^ T cell recognition of the naturally occurring MHC-I-restricted LCMV-associated immune escape variant Y4F is restored following vaccination with a proline-altered peptide ligand (APL). The APL increases MHC/peptide (pMHC) complex stability, rigidifies the peptide and facilitates T cell receptor (TCR) recognition through reduced entropy costs. Structural analyses of pMHC complexes before and after TCR binding, combined with biophysical analyses, revealed that although the TCR binds similarly to all complexes, the p3P modification alters the conformations of a very limited amount of specific MHC and peptide residues, facilitating efficient TCR recognition. This approach can be easily introduced in peptides restricted to other MHC alleles, and can be combined with currently available and future vaccination protocols in order to prevent viral immune escape.

## Author summary

Viral escape mutagenesis correlates often with disease progression and represents a major hurdle for vaccination-based therapies. Here, we have designed and developed a novel generation of altered epitopes that re-establish and enhance significantly CD8^+^ T cell recognition of a naturally occurring viral immune escape variant. Biophysical and structural analyses provide a clear understanding of the molecular mechanisms underlying this reestablished recognition. We believe that this approach can be implemented to currently available or novel vaccination approaches to efficiently restore T cell recognition of virus escape variants to control disease progression.

## Introduction

Recognition of major histocompatibility complex class I (MHC-I)-restricted viral peptides is a prerequisite for CD8^+^ T-cell activation, control and/or clearance of viral infections. Usually, cytotoxic T-lymphocyte (CTL) responses are directed towards a limited number of immunodominant viral peptides [1] and selection pressure imposed by adaptive immune responses leads often to the emergence of viral populations with a limited number of recurring escape mutations [2–4]. Epitope mutations can impair CTL responses [5] by *e*.*g*. altering antigen processing [6, 7], reducing the overall stability of peptide/MHC complexes (pMHC) [8, 9] and/or disrupting T-cell receptor (TCR) recognition [10, 11]. CTL escape variants correlate with disease progression [12, 13] and represent a major hurdle for disease control as well as for the design of T-cell based vaccines [14].

To our knowledge, previous use of wild-type and escape epitopes in vaccination experiments has not provided efficient CTL responses against MHC-restricted viral escape variants [14, 15]. Therefore, the design of altered peptide ligands (APLs) that could promote such responses would represent a crucial step towards the development of efficient vaccines [16]. Although our understanding of the interactions between TCRs and pMHC has increased dramatically, the impact of individual peptide modifications on TCR recognition remains difficult to predict. Even subtle peptide alterations can significantly impact on pMHC stability, and impair or abolish T cell recognition. A conventional and sometimes successful approach to design APLs with enhanced pMHC stability and immunogenicity has been to optimize interactions between peptide anchor residues and MHC binding pockets [17–19]. However, escape variants that target TCR recognition often exhibit optimal MHC anchor residues, reducing possibilities for such modifications. Optimally, the introduced modifications should also not alter the conformation of APLs compared to the original epitopes, in order to elicit efficient cross-reactive CTL responses towards the wild-type epitope [18, 20, 21]. Therefore, the design of a novel generation of APLs that could promote such responses would represent a crucial step towards the development of efficient anti-viral T-cell based vaccines [22].

We have previously demonstrated that the immunogenicity of the cancer-associated H-2D^b^-restricted antigen gp100_25-33_ [23] or the T cell epitope associated with impaired peptide processing (TEIPP) neo-epitope Thr4 [24–26] was dramatically improved following substitution of peptide position 3 to a proline (p3P). Comparative structural analyses revealed that the conformation of the APLs was similar to wild-type epitopes, and that the stabilizing effect of p3P is accounted for by van der Waals and CH-π interactions with the H-2D^b^ residue Y159, conserved among most known mouse and human MHC-I alleles, resulting in rigidification of the pMHC complex [27]. Importantly, vaccination with p3P-modified APLs elicited high frequencies of CTLs from the endogenous repertoire that efficiently targeted H-2D^b^/gp100_25-33_ and H-2D^b^/Trh4 complexes on melanoma cells [23, 24].

In the present study, we addressed if we could restore endogenous T cell recognition of a naturally occurring viral escape variant following vaccination with a p3P-modified APL. It is well established that infection of C57/Bl6 mice with lymphocytic choriomeningitis virus (LCMV) induces robust CTL responses towards the immunodominant H-2D^b^-restricted epitope gp33 (KAV**Y**NFATM) [28]. Upon CTL pressure, a limited number of mutations in gp33 emerge, with consistent patterns, allowing for viral CD8^+^ T-cell escape [2, 4, 29, 30]. The main naturally occurring mutation that allows LCMV to efficiently escape immune recognition, is the Y4F substitution (KAV**F**NFATM) which abrogates endogenous CD8^+^ T cell recognition as well as recognition by the H-2D^b^/gp33-specific TCR P14. Here, we demonstrate that peptide vaccination with V3P_Y4F (KA**PF**NFATM) restores P14 recognition of Y4F in LCMV-infected mice. Furthermore, vaccination with influenza constructs that encode for V3P_Y4F provokes significant endogenous CD8^+^ T cell cross-recognition of Y4F. Comparison of crystal structures of an ensemble of pMHC complexes before and after binding to the TCR P14 revealed that i) P14 binds nearly identically to all complexes, ii) the conformations of peptide residues p1K and p6F as well as H-2D^b^ residues R62, E163 and H155 are affected by the p3P modification, predisposing pMHC complexes for enhanced TCR recognition. The p3P modification also decreases the entropic penalty for TCR recognition. In conclusion, our results demonstrate the possibility to vaccinate with modified peptides and/or proteins for enhanced T cell recognition, and may form an alternative basis for novel strategies to target viral escape mutants.

## Results

### The p3P modification enhances pMHC stability without altering structural conformation, restoring P14 TCR recognition

**Fig 1:**
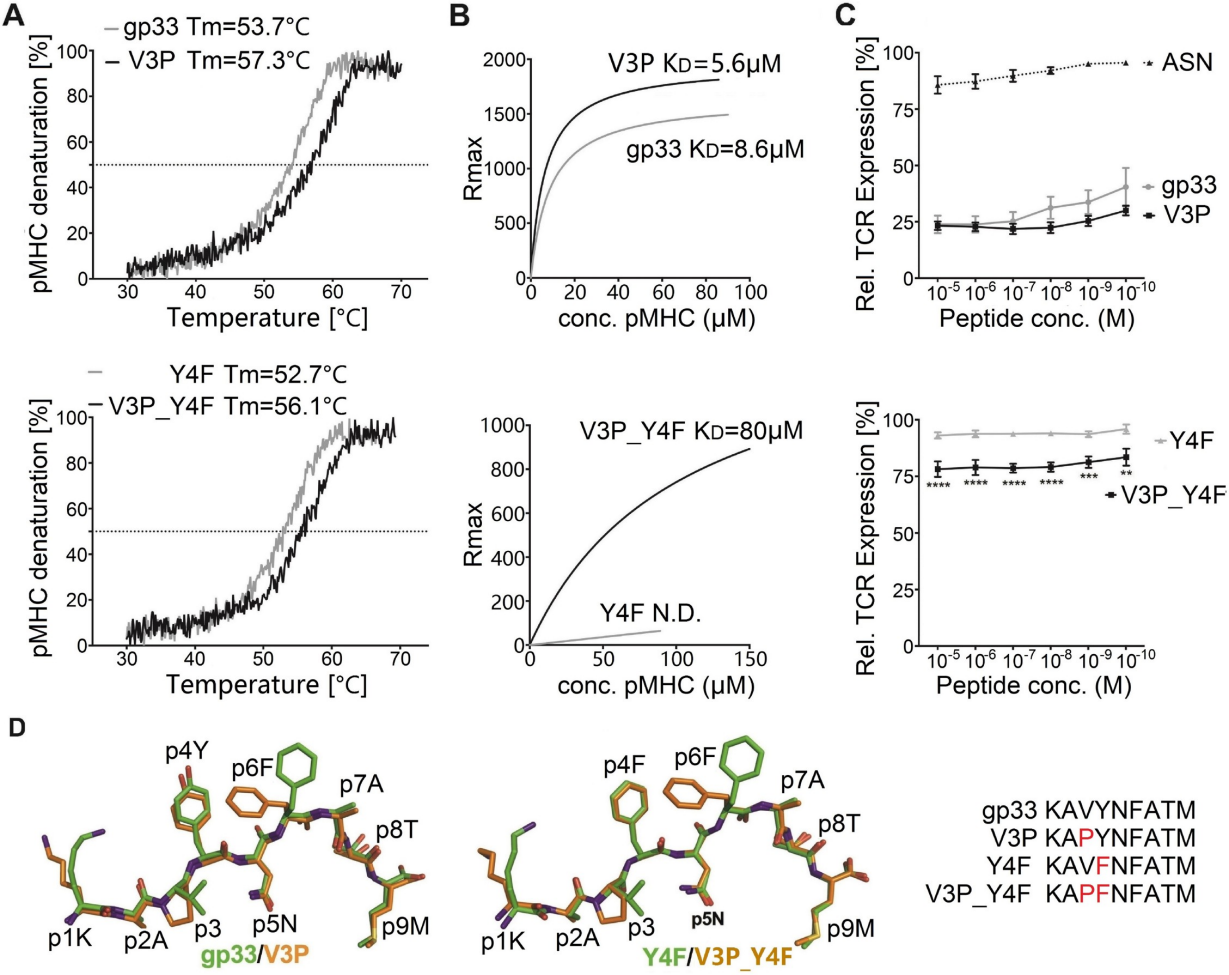
The p3P modification enhances pMHC stability without altering structural conformation, reestablishing TCR recognition. **A. The p3P modification increases pMHC stability.** CD melting curves of H-2D^b^/gp33 and H-2D^b^/V3P (upper panel), and H-2D^b^/Y4F and H-2D^b^/V3P_Y4F (lower panel). Melting temperatures (T_m_) corresponding to 50% protein denaturation are indicated. **B. The soluble TCR P14 binds to the APL V3P_Y4F.** In contrast to Y4F, V3P_Y4F is bound by P14. Binding affinity of the soluble TCR P14 to each pMHC was measured using SPR. K_D_ values are indicated. **C. The p3P modification increases TCR internalization.** TCR downregulation was measured following exposure of P14 T cells to H-2D^b^ in complex with each peptide at indicated concentrations on RMA cells. CD3^+^CD8^+^CD4^-^ and Vα2^+^ cells were gated to quantify TCR internalization and p values calculated by using two-way Anova with Turkey’s multiple comparison test. **** represents p<0.0001; *** 0.0002 and ** 0.0018. The H-2D^b^-restricted Influenza-derived peptide ASNENMETM (ASN) was used as negative control. **D. The p3P modification does not alter the conformation of the backbone of APLs compared to native counterparts.** Superposition of the crystal structures of H-2D^b^/V3P and H-2D^b^/V3P_Y4F with H-2D^b^/gp33 and H-2D^b^/Y4F demonstrates that the p3P modification does not alter backbone conformations. Significant conformational changes are only observed for the side chains of peptide residues p1K and p6F following the p3P substitution.

Circular dichroism (CD) measurements revealed a consistent increase in pMHC complex thermal stability for the p3P-substituted peptides V3P (KAPYNFATM) and V3P_Y4F (KAPFNFATM) compared to the wildtype gp33 (KAVYNFATM) and escape variant Y4F (KAVFNFATM) epitopes, respectively (Fig 1A, Table 1). Importantly, H-2D^b^/gp33 and H-2D^b^/Y4F display equivalent thermal stability (Fig 1A). Furthermore, surface plasmon resonance (SPR) analyses revealed significantly higher binding affinity of soluble P14 TCR to H-2D^b^/V3P compared to H-2D^b^/gp33 and significantly higher binding affinity of soluble P14 TCR to H-2D^b^/V3P_Y4F compared to H-2D^b^/Y4F (Fig 1B, Table 1). In contrast to an undetectable affinity to H-2D^b^/Y4F, P14 bound to H-2D^b^/V3P_Y4F. Interactions between soluble P14 and H-2D^b^/gp33 and H-2D^b^/V3P were also characterized using isothermal titration calorimetry (ITC), revealing that binding of P14 to H-2D^b^/V3P was mainly enthalpy-driven with almost null contribution of entropy, whereas binding to H-2D^b^/gp33 was entropically unfavorable (S1 Fig, Table 1). In conclusion, the p3P modification increases pMHC stability, resulting in recognition of V3P_Y4F by P14 and enhances TCR affinity by decreasing the entropic cost for binding.

**Table 1:** pMHC stability, P14 TCR affinity and thermodynamic parameters of gp33 variants.

Peptide	gp33	V3P	Y4F	V3P_Y4F
Sequence	KAVYNFATM	KA**P**YNFATM	KAV**F**NFATM	KA**PF**NFATM
T_m_ (C)^a^	53.7±0.1	57.3±0.4	52.7±0.4	56.1±1.1
K_D_ (μM)^b^	8.6±0.4	5.6±0.4	ND	80.3±22
K_D_ (μM)^c^	5.8±2.4	1.1±0.2	-	-
TΔS (kcal/mol)^c^	-3.1±1.0	-0.5±0.3	-	-
ΔH (kcal/mol)^c^	-10.2±0.7	-8.5±0.5	-	-

^a^ Measured with Circular Dichroism where T_m_ is the temperature with 50% pMHC denaturation.

^b^ Determined from steady state SPR data with software BIAevaluation.

^c^ Measured using ITC200 at 25C. ND–Not Determinable. The values are an average of at least two independent experiments ±SD

Next, P14 TCR down-regulation was assessed upon exposure to gp33, V3P, Y4F or V3P_Y4F-loaded H-2D^b+^ RMA cells (Fig 1C). While H-2D^b^/gp33 induced significant TCR down-regulation, minimal TCR downregulation was detected with Y4F, even at high peptide concentrations. Exposure of P14 T cells to V3P equaled or increased TCR internalization compared to gp33. Importantly, exposure to V3P_Y4F increased P14 TCR down-regulation compared to Y4F (Fig 1C). The crystal structures of H-2D^b^/V3P and H-2D^b^/V3P_Y4F were determined to 2.5 and 2.6 Å resolution (S1 Table), and compared with H-2D^b^/gp33 [3] and H-2D^b^/Y4F [2] (Fig 1D, S2 Fig). The overall structures of all pMHCs are nearly identical, and the amount of hydrogen bond and van der Waals interactions formed between H-2D^b^ and each p3P-APL was equivalent to each wild-type epitope counterpart. The backbone of the p3P-APL corresponds exactly to the wild-type peptides, indicating that the p3P modification does not alter the conformation of APLs compared to wild-type peptides (Fig 1D). The root mean square deviation values for main chain atoms are 0.24–0.67 Å^2^ and 0.20–0.24 Å^2^ for the backbone of the H-2D^b^ heavy chain and the peptides. The only significant conformational differences between wild-type and p3P-APLs were side chain movements of peptide residues p1K and p6F towards the N-terminal and middle section of the peptide-binding cleft of H-2D^b^ (Fig 1D, S2 Fig).

### In contrast to Y4F, V3P_Y4F induces significant P14 T cell responses both *in vitro* and *in vivo*

First, we assessed the functional effects of all peptides on P14 T-cell activation by comparing intracellular TNF and IFNγ production, T cell degranulation (CD107a) as well as target cell lysis. CD8^+^ T cells, isolated from spleens of naïve or gp33-immunized P14 transgenic mice (P14-tg), were co-cultured with RMA cells pulsed with each peptide. Peptides gp33, V3P and V3P_Y4F induced significant production of TNF and IFNγ, as well as CD107a expression, while Y4F failed to induce any P14 T cell response (S3 Fig). Lysis of RMA cells by P14 T cells was also enhanced with V3P_Y4F compared to Y4F (S3 Fig). In conclusion, p3P-modification of the immune escape variant Y4F re-establishes *in vitro* recognition by P14 T cells (S3 Fig).

**Fig 2:**
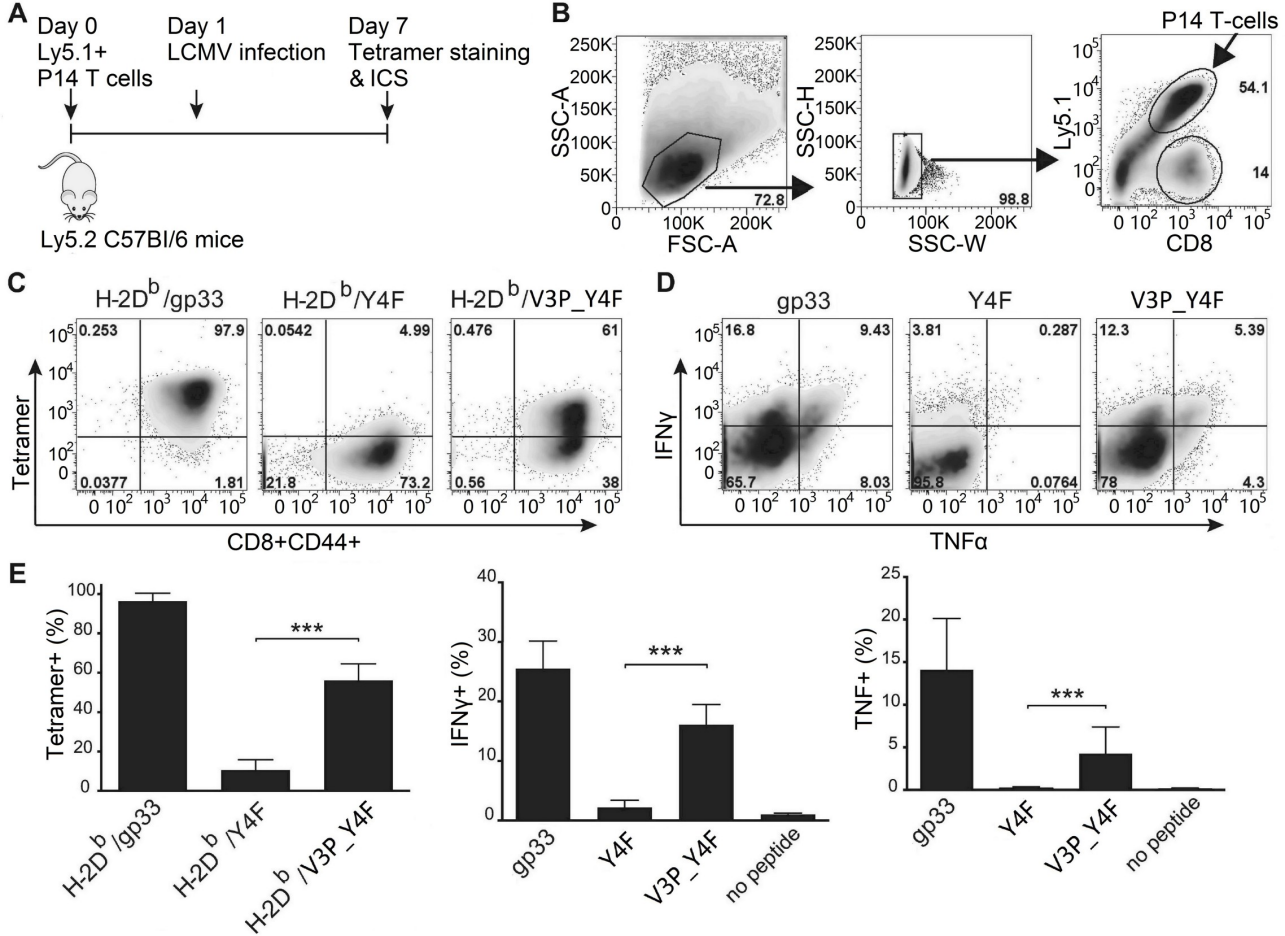
The p3P modification increases significantly P14 T cell responses. **A.** C57/Bl6 mice were adoptively transferred with 10^4^ P14 T-cells one day prior to infection with LCMV. Mice were sacrificed on day 7 post T cell transfer. T-cells from spleen were stained with PE-conjugated H-2D^b^/gp33, H-2D^b^/Y4F or H-2D^b^/V3P_Y4F tetramers. T cells were also stimulated with gp33, Y4F or V3P_Y4F peptides (10^−6^ M) for 5h, prior to assessment of intracellular IFNγ and TNF expression levels. **B.** Gating strategy used to detect CD8^+^ CD44^+^ cells. P14 T cells were distinguished from endogenous T-cells using the Ly5.1 (V450) marker. **C.** Representative density plots from tetramer staining. CD8^+^ CD44^+^ P14 T-cells were stained with H-2D^b^/gp33 (left), H-2D^b^/Y4F (middle) and H-2D^b^/V3P_Y4F (right) tetramers. **D.** Representative ICS density plots. P14 T-cells were stimulated with peptides gp33 (left), Y4F (middle) or V3P_Y4F (right). **E.** CD8^**+**^ CD44^+^ P14 T-cells from the spleen were stained with the indicated tetramers on the x-axis (left). P14 T-cells from the spleen were stimulated with the peptides indicated on the x-axis, and expression of INFγ (middle) and TNF (right) was assessed. Error bars show mean +/- SD. One-way Anova was performed to compare between different groups. P-values * and *** represent p<0.05 and p<0.001. The analysis was made using the GraphPad Prism software.

We thereafter assessed the *in vivo* impact of the p3P modification on LCMV-activated P14 T cells. 10^4^ P14 T-cells were adoptively transferred into C57Bl/6 mice, thereafter infected with the LCMV clone 13 (Fig 2). Six days post-infection, P14 T-cells isolated from spleens (Fig 2A and 2B) were either stained with pMHC tetramers or re-stimulated with 10^−6^ M gp33, Y4F or V3P_Y4F. Tetramer staining demonstrated that a significant amount of the activated P14 T cells recognized the H-2D^b^/V3P_Y4F complex (Fig 2C–2E). Furthermore, while V3P_Y4F- and gp33-stimulated P14 T-cells produced TNF and IFNγ, Y4F was not recognized (Fig 2D and 2E). Altogether, these results demonstrate that, in contrast to Y4F, V3P_Y4F is efficiently recognized by P14 T cells *in vivo*-activated by LCMV infection.

### Vaccination with Influenza encoding for V3P_Y4F activates endogenous CD8^+^ T-cells that cross-react and recognize the immune escape variant Y4F

**Fig 3:**
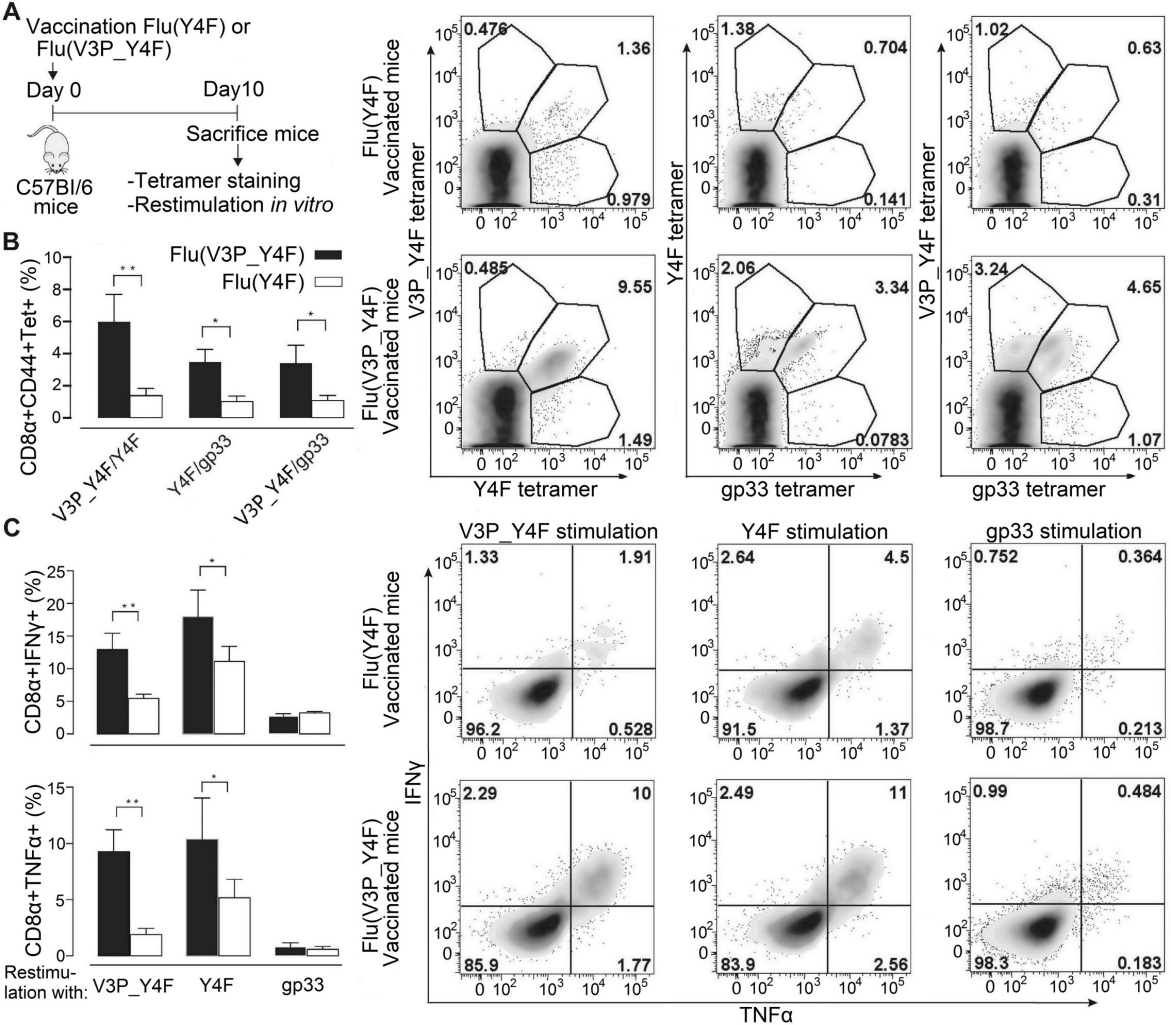
Vaccination of C57/Bl6 mice with influenza virus encoding for V3P_Y4F re-established efficient recognition of the immune escape variant Y4F. The escape mutant Y4F (KAV**F**NFATM) and the proline-modified variant V3P_Y4F (KA**PF**NFATM) were engineered into the stalk region of neuraminidase of the Influenza A virus strain HKx31 (H3N2), and used to infect C57BL/6 mice. **A.** C57/Bl6 mice infected with either flu(Y4F) or flu(V3P_Y4F) were sacrificed day 10 post infection. **B.** CD8^+^ CD44^+^ cells were stained with combinations of H-2D^b^/gp33, H-2D^b^/Y4F or H-2D^b^/V3P_Y4F tetramers. Data represents double positive tetramer populations. Right top panel: Representative density plots of CD8^+^ CD44^+^ T-cells from mice infected with flu(Y4F) or flu(V3P_Y4F). Data from pooled 4–5 mice, representative of two different experiments. **C.** Cells were also stimulated with gp33, Y4F or V3P_Y4F peptides for 5 h, and intracellular IFNγ and TNF expression was determined. (Right bottom panel) CD8^+^ CD44^+^ T-cells isolated from mice infected with flu(Y4F) or flu(V3P_Y4F) were stimulated with either gp33, Y4F or V3P_Y4F (10^−6^ M), and thereafter stained for INFγ and TNF. Data of IFNγ and TNF secretion from pooled 4–5 mice representative of two different experiments. Error bars show mean +/- SD. Statistical significance is presented with the p-value from a two-way Anova with Sidak’s multiple comparison test. * represents p<0.05; ** represents p<0.01. The analyses were performed using the GraphPad Prism software.

Next, we assessed if vaccination with V3P_Y4F could elicit endogenous T cells that cross-react and recognize Y4F. We engineered Y4F and V3P_Y4F into the stalk region of the Influenza A Neuraminidase (HKx31). This well-established model results in efficient processing and presentation of epitopes on infected cells [31]. C57/Bl6 mice were infected with the modified viruses Flu(Y4F) or Flu(V3P_Y4F) (Fig 3A). 10 days following infection, CD8^+^CD44^+^ splenocytes (S4 Fig) were co-stained with combinations of H-2D^b^/gp33(PE)-H-2D^b^/Y4F(APC) tetramers, H-2D^b^/gp33(PE)-H-2D^b^/V3P_Y4F(APC) tetramers or H-2D^b^/Y4F(PE)-H-2D^b^/V3P_Y4F(APC) tetramers in order to detect cross-reactive T cell populations (Fig 3B and 3C). Vaccination with Flu(V3P_Y4F) elicits endogenous T cell populations that bind to both H-2D^b^/Y4F and H-2D^b^/V3P_Y4F tetramers equally well (Fig 3B). Interestingly, Flu(V3P_Y4F) vaccination also elicits endogenous T cell populations with dual specificity to H-2D^b^/gp33 and H-2D^b^/Y4F tetramers. In contrast, H-2D^b^/gp33-, H-2D^b^/Y4F- and H-2D^b^/V3P_Y4F-tetramer staining after vaccination with Flu(Y4F) failed to identify any significant T cell population (Fig 3B).

Intracellular expression of IFNγ and TNF in CD8^+^CD44^+^ endogenous T cells was assessed following stimulation with peptides gp33, Y4F or V3P_Y4F (Fig 3C). In contrast to Flu(Y4F), vaccination with Flu(V3P_Y4F) results in significantly enhanced IFNγ and TNF levels towards both Y4F and V3P_Y4F. However, vaccination with neither Flu(Y4F) nor Flu(V3P_Y4F) induced any elicitation of IFNγ and TNF towards gp33. This is well in line with previous studies in which the Y4F-specific T cell clone YF.F3 killed efficiently targets presenting gp33 but did not produce IFNγ [32]. Similar results were obtained using bronchoalveolar lavage (BAL)-derived T cells (S4 Fig). In conclusion, vaccination with Flu(V3P_Y4F) induces endogenous T cell populations that respond strongly to H-2D^b^/V3P_Y4F and efficiently cross-react with H-2D^b^/Y4F.

### The T cell receptor P14 binds identically to H-2D^b^/gp33, H-2D^b^/V3P and H-2D^b^/V3P_Y4F

In order to assess the molecular bases underlying the effects of the p3P modification on T cell recognition, we determined the crystal structures of the ternary complexes P14/H-2D^b^/gp33, P14/H-2D^b^/V3P and P14/H-2D^b^/V3P_Y4F to 3.2, 2.8 and 1.75 Å resolution (S2 Table, S5 Fig). All ternary complexes are almost identical with rmsd values of 0.4Å, 0.1–0.3Å, 0.3–0.4Å and 0.3–0.4Å for peptide clefts, peptides, TCRα and TCRβ. The three ternary complexes displayed a typical TCR/pMHC binding mode with P14 diagonally positioned over the pMHC complexes (S6 Fig). The ternary structures revealed very similar TCR contacts with H-2D^b^ presenting the three different peptides, with identical conformations of the six P14 CDR loops (S6 Fig). Although CDR3α (101-**Y**G**NE**K-105) and CDR3β (93-**D**AG**GR**NTL-100) are located over the middle part of each peptide variant, only CDR3β forms hydrogen bonds with the three peptide residues p4Y, p6F and p8T (S7 Fig). All the other P14 loops CDR1α (33-E**D**S**T**F**N**-38), CDR1β (25-NNH**D**YM-30), CDR2α (58-**L**S**V**S-61) and CDR2β (46-YS**Y**-48) interact with the H-2D^b^ heavy chain (S7 Fig).

### The immune escape mutation Y4F abrogates the hydrogen bond network formed with P14

The P14 CDR3β residues D93, G96 and R97 form a network of hydrogen bonds with the side chains of the gp33 residues p4Y and p8T, as well as with the backbone of p5N and p8T (S7 Fig). The side chain of R97β runs parallel with the peptide, stretching out to reach to the tip of p4Y, forming van der Waals interactions with the side chain of p6F, forcing its rotation in the case of gp33. The TCR residue Y101α, which side chain is positioned between p1K and p4Y, forms a hydrogen bond with the H-2D^b^ residue E163, which also forms a hydrogen bond with p4Y (S7 Fig). Thus, the hydroxyl group of p4Y plays a key role in a net of hydrogen bond and van der Waals interactions formed with TCR residues N38α and Y101α as well as the H-2D^b^ residue E163. The introduction of the Y4F mutation will abrogate all these interactions, abolishing P14 recognition (S7 Fig). Furthermore, the Y4F mutation should introduce higher hydrophobicity within this key TCR/pMHC interface, composed mainly by polar residues. Altogether, this explains why P14 does not bind nor recognize the immune escape variant H-2D^b^/Y4F.

### The p3P modification facilitates TCR recognition

**Fig 4:**
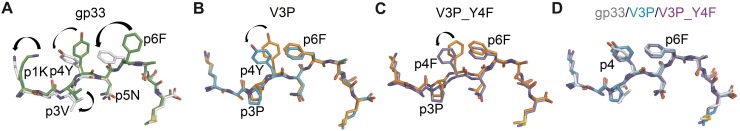
The p3P modification results in conformational changes of peptide residues p1K and p6F, predisposing pMHCs for optimal binding to P14. **A.** Comparison of gp33 before binding (in green) and after binding (in white) to P14 reveals major conformational changes in gp33 following binding to P14. These include a movement of the p2-p4 backbone of gp33 that is pushed down in the cleft combined with a 180 degrees rotation of the isopropyl moiety in residue p3V. Furthermore, the side chain of peptide residues p1K, P4Y and p6F all take new conformations following binding to P14. All movements are indicated by blue arrows. **B.** The introduction of p3P in V3P results in optimal positioning of the side chains of residues p1K and p6F prior to binding to P14 (in orange). The only observed conformational difference was taken by residue p4Y following V3P binding to P14 (in cyan). **C.** Similarly to V3P, the only conformational difference observed for V3P_Y4F before (in orange) and after (in violet) binding to P14 is at peptide residue p4Y. **D.** Peptides gp33 (in white), V3P (in cyan) and V3P_Y4F (in violet) take nearly identical conformations when bound to P14.

The three ternary TCR/MHC/peptide structures were compared with each corresponding TCR-unbound pMHC (Fig 4, S8 Fig). The side chain of p4Y, essential for recognition by P14 [4, 33, 34], rotates down following P14 binding to both H-2D^b^/gp33 and H-2D^b^/V3P (Fig 4A and 4B). A similar rotation was also observed for residue p4F in H-2D^b^/V3P_Y4F upon binding to P14 (Fig 4C). The side chain of p6F in gp33 is also affected upon binding to P14 (Fig 4A). Interestingly, the p3P modification resulted in a similar conformation for p6F in both H-2D^b^/V3P and H-2D^b^/V3P_Y4F prior to binding to P14 (Fig 1D and Fig 4). Furthermore, the side chain of residue p1K in H-2D^b^/gp33 also moves towards the N-terminal of the peptide binding cleft following P14 binding (Fig 4A), taking an identical conformation as in both p3P-substituted peptides (Fig 4D).

One of the most significant differences in H-2D^b^/gp33, before and after binding to P14, is a shift of the p2-p4 backbone of gp33 when bound to P14, towards the binding cleft of H-2D^b^. Following P14 docking, p3V in gp33 extends 1.2 Å deeper into the D-pocket of H-2D^b^, combined with a 180° rotation (Fig 4A). In contrast to gp33, the p2-p4 section is more constrained in both V3P and V3P_Y4F, following TCR binding (Fig 4B and 4C). However, it should be noted that the final conformations of all three peptides in the ternary complexes is nearly identical (Fig 4D). In conclusion, residues 1 and 6 in p3P-APLs take the same conformations prior to TCR binding as found in the ternary complexes, potentially enabling a more favorable surface for P14 TCR binding.

**Fig 5:**
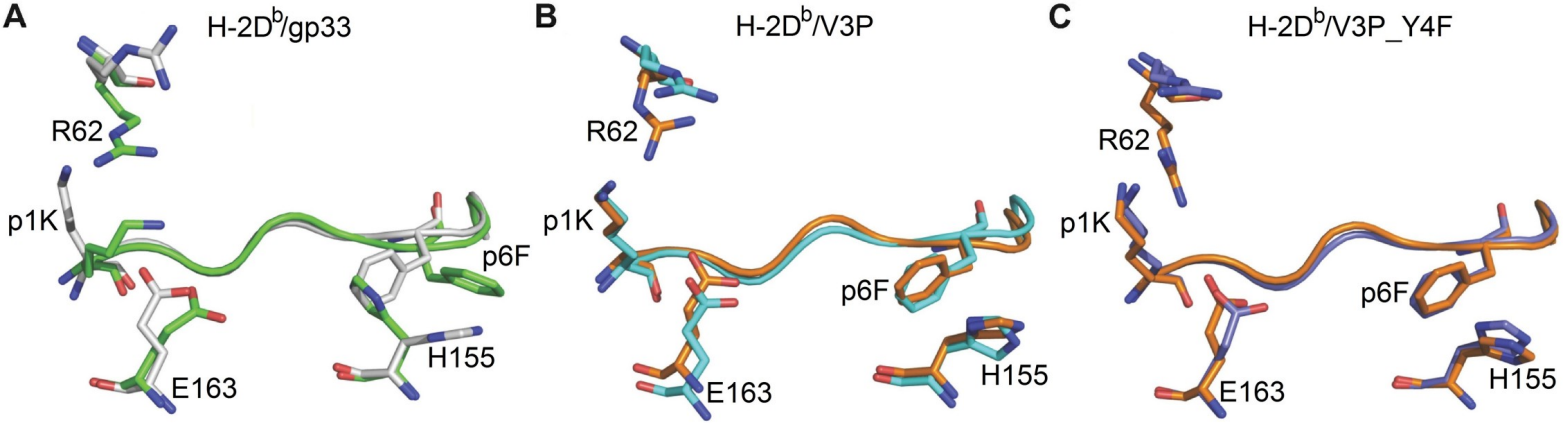
The p3P modification affects the conformations of peptide residues p1K and P6F, as well as H-2D^b^ residues R62, H155 and E163 facilitating TCR recognition. **A.** Comparison of H-2D^b^/gp33 before (in green) and after P14 binding (in white) reveals that the conformation of a very limited amount of pMHC residues is affected (shown as sticks). Following binding to P14, the side chain of peptide residue p1K moves towards the N-terminal part of the peptide binding cleft while the side chain of p6F rotates. As a consequence, conformational changes are observed only for heavy chain residues R62, H155 and E163. **B.** In contrast to gp33, the introduced p3P modification already positions most peptide and heavy chain residues in optimal conformations, limiting significantly the required movements following binding to P14. pMHC residues before and after binding to P14 are colored orange and cyan. **C.** Similarly to V3P, the p3P modification in V3P_Y4F results in optimal positioning of all key peptide and heavy chain residues prior to binding to P14. pMHC residues before and after binding to P14 are colored orange and violet.

The crystal structures of TCR-unbound and TCR-bound pMHCs also revealed that conformational differences in H-2D^b^ residues were observed only for residues R62, E163 and H155 (Figs 5, S2, S9 and S10). The large movement of p6F in gp33 following binding to P14 induces the counter wise reorientation of the side chain of residue H155 towards the TCR (Fig 5A). The redisposition of H155 and p6F in H-2D^b^/gp33 promotes the adequate positioning of the key TCR residue R97β, which runs longitudinally along the length of the N-terminal part of the peptide (S7 Fig). In contrast, residues p6F and H155 are already optimally positioned in both the TCR unbound and bound forms of the H-2D^b^/V3P and H-2D^b^/V3P_Y4F complexes (Fig 5B and 5C), most probably predisposing for optimized interactions with P14.

Furthermore, p1K in gp33 also takes a different conformation upon binding to P14, bending backwards towards the H-2D^b^ residues R62 and E163, which conformations are affected (Fig 4A, Fig 5A). Here again, the side chain of p1K takes exactly this conformation in both V3P and V3P_Y4F already before TCR binding (Fig 4, Fig 5). Altogether, p1K, P6F and heavy chain residues R62, H155 and E163 have already adopted in the unbound V3P and V3P_Y4F complexes similar conformations to those observed in all three ternary structures (Fig 4 and Fig 5). Thus, the p3P substitution potentially facilitates TCR recognition by positioning specific key peptide and MHC residues prior to the formation of the ternary complexes. This is well in line with our SPR and ITC results, which indicate that the energy required for P14 recognition of V3P is reduced compared to gp33 (Table 1, S1 Fig).

## Discussion

Subsets of peptide analogs have been used by others and us to both break T cell tolerance and enhance T cell responses to tumors [16, 23, 35]. Heteroclitic subdominant viral T cell determinants were also used to enhance both pMHC stability and T cell avidity towards the mouse hepatitis virus-specific subdominant S598 determinant [22, 36]. Most, if not all studies performed in other laboratories have focused their efforts on introducing peptide mutations that would significantly increase the stability of pMHCs with as little alteration as possible of peptide conformation. Here, instead of mutating a key anchor position, we targeted interactions between peptide position 3 and the MHC residue Y159, conserved among most known mouse and human alleles. Indeed, besides H-2D^b^, we have previously demonstrated that the p3P modification enhances significantly the stability of H-2K^b^ in complex with different TAAs [23]. Thus, the p3P modification could potentially enhance stabilization of other MHC-I alleles that comprise the heavy chain residue Y159, leading to enhanced TCR recognition.

Here, we addressed if we could increase CD8^+^ T cell avidity and restore recognition of the viral escape variant Y4F that binds to H-2D^b^ with the same high affinity as gp33 [37]. The TCR P14 is specific for H-2D^b^/gp33 and it has been previously demonstrated that P14 recognition is abolished by the Y4F mutation [2, 3]. Comparison of the crystal structures of H-2D^b^/gp33 and H-2D^b^/Y4F demonstrated that the only structural difference between these two pMHCs was the removal of the hydroxyl tip from the peptide residue p4 [2]. We demonstrate that the p3P modification in V3P_Y4F overcomes the restrictions imposed by the Y4F mutation, reestablishing P14 recognition of this structural mimic of Y4F. Furthermore, we show that it is fully possible to restore endogenous CD8^+^ T cell recognition of Y4F following vaccination with V3P_Y4F. Possibly, the higher avidity of subsets of the endogenous T cell population for H-2D^b^/V3P_Y4F pushes them over a certain threshold of activation, and the molecular similarities between H-2D^b^/V3P_Y4F and H-2D^b^/Y4F allow for cross-reactivity, resulting in significant cytokine secretion towards Y4F. However, *in vitro* re-stimulation with gp33 of endogenous CD8^+^ T cells isolated from Flu(V3P_Y4F)-vaccinated mice did not induce any significant secretion of cytokines, although these endogenous CD8^+^ T cells recognized both V3P_Y4F and gp33-loaded MHC tetramers. Martin *et al* have previously provided evidence for selective activation of different effector functions in CD8^+^ T cells by APLs. More specifically, the results of their study show that the H-2D^b^/Y4F-specific T cell clone YF.F3 killed efficiently targets presenting gp33 but did not produce high amounts IFNγ against gp33 [32]. This is well in line with the results presented in this study. Altogether, this suggests to us that vaccination with a cocktail of epitopes could provide wider protection against both immunodominant and immune escape targets.

So how does it possibly work at the molecular level? The rigidification of p3P-modified peptides could enhance TCR recognition by decreasing entropic costs. Indeed, we have previously demonstrated in TAA models that peptide rigidification enhanced considerably TCR recognition [26, 27]. Overall the effects of proline replacement on protein stability and function are well established for a large ensemble of proteins [38], revealing that protein-protein interactions often occur through regions enriched with proline residues [39]. Proline substitutions increase overall protein stability as well as the stability of specific protein regions [40]. Indeed, proline replacement of specific residues in TCR CDR loops can increase significantly recognition of antigens [41]. The importance of the interaction of peptide residue p3P with residue Y159, conserved amongst most known MHC-I alleles, has been previously described [27], revealing that p3P reduces significantly the flexibility of the pMHC complex, thus decreasing unfavorable entropic change upon complex formation. Such reduced entropic penalties for TCR recognition following p3P mutation were confirmed here by ITC measurements, which indicated reduced unfavorable entropic contribution for recognition of H-2D^b^/V3P by P14 compared to H-2D^b^/gp33. The importance of the reduction of peptide conformation heterogeneity for enhanced TCR has been described, using a combination of crystal structure and molecular dynamic studies [42]. A peptide that must move to optimize the interactions with the bound TCR will increase the entropic cost for binding, resulting in slower binding, lower affinity and less efficient recognition [43]. Consequently, although many TCRs bind with unfavorable entropy changes [37, 44], reduction of conformational heterogeneity coupled with rigidification of the peptides may lead to enhanced T cell recognition. In this study, the p3P mutation reduces motion and therefore enhances T cell recognition by increasing T cell association rate and decreasing entropic costs for binding.

Although X-ray structural studies of proteins provide accurate snapshots of protein complexes, crystal structures provide relatively little information about the dynamic bases underlying protein-protein interactions. The dynamic motions of both pMHC and TCR impact on recognition by T cells, clearly influencing function and recognition [42]. Here, we compared the crystal structures of each studied pMHC complex before and after P14 TCR binding (besides the P14/H-2D^b^/Y4F complex that could not be obtained since P14 does not bind to this pMHC). Peptides tune the motions of MHC heavy chains and reduced motions may lead to enhanced recognition. Besides the peptide rigidification imposed by the p3P modification, comparison of a structural snapshot for each ternary structure with each TCR-unbound pMHC variant indicated an additional structural reason for the increased TCR recognition of p3P-modified epitopes. In all cases, conformational differences were observed in peptide residues p1K and p6F in V3P_Y4F and V3P, compared to Y4F and gp33, before TCR binding. In all p3P cases, the side chains of peptide residues p1K and p6F took the same conformation, as observed in the ternary structures, prior to TCR binding. In line with this, others [33, 45] and we [37] have previously demonstrated the importance of residue p1K for recognition by the TCR P14. The crystal structure of the semi-agonist Y4A (KAVANFATM) also revealed a similar conformation for both p1K and p6F prior to binding to P14 TCR [37]. Furthermore, the conformation of the MHC “TCR footprint” heavy chain residues R62, H155 and E163 [46, 47] was also affected following p3P substitution, possibly due to the movements of p1K and p6F. Altogether, prior to TCR landing, the p3P modification alters the conformation of residues both in the peptide and the MHC heavy chain similar to conformations taken upon binding to the TCR, thus predisposing the pMHC for facilitated TCR recognition.

The results presented within this study indicate in our opinion that i) docking of P14 to p3P-modified peptides is facilitated since the conformations of key residues in both peptide and heavy chain are already optimal prior to TCR binding (ready-to-go conformation); ii) consequently, the energetic costs for TCR recognition should be reduced since there is no need for any major movement in the rigidified epitope besides the conformational change for residue p4Y. As vaccination with V3P_Y4F restored endogenous T cell recognition of Y4F, the p3P modification could thus represent a novel way to increase the immunogenicity of a large array of H-2D^b^-restricted epitopes as well as possibly viral epitopes restricted by other MHC alleles. We thus describe here a successful approach to restore recognition of viral escape peptide that can be easily coupled to already existing vaccination protocols, including vaccination with full-length proteins as well as *e*.*g*. modified mRNA vaccines, by introducing the p3P modification in a selection of viral epitopes.

## Materials and methods

### Cell lines and mice

H-2D^b+^/H-2K^b+^ RMA cells, kindly provided by Prof. Klas Kärre, were used as target cells in the functional assays described below. Pathogen-free wild-type (WT) C57BL/6 (B6) and RAG1/2-deficient (RAG1/2^-/-^) P14-transgenic mice were bred and maintained within the facilities of the MTC department, Karolinska Institute. Vα2^+^ T cells from P14 mice were used as effector cells for *in vitro* experiments. P14 mice were used for *in vivo* T-cell stimulation assays.

### Peptides and antibodies

Peptides gp33, Y4F, V3P and V3P_Y4F as well as the control peptide NP_366_ (ASNENMETM, abbreviated as ASN) were purchased from GenScript (Piscataway, NJ, USA). Antibodies 53–6.7 (anti-CD8α), 53–5.8 (anti-CD8β), XMG1.2 (anti-IFN-γ), MP6-XT22 (anti-TNF), 145-2C11 (anti-CD3ε), 1D4B (anti-CD107a), BP-1 (anti-Ly5.1/CD249), IM7 (anti-CD44) and B20.1 (anti-TCR Vα2) were purchased from BD Biosciences (San Diego, CA, USA). Antibodies GK1.5 (anti-CD4) and H57-597 (anti-TCR Cβ) were purchased from Abcam (Cambridge, UK) and eBioscience (San Diego, CA, USA).

### Preparation, refolding and crystallization of TCR/pMHC complexes

Refolding of all pMHCs was conducted as previously described [48]. P14 was produced and refolded by dilution and thereafter-purified using ion exchange and size exclusion chromatography. Crystals for H-2D^b^/V3P and H-2D^b^/V3P_Y4F were obtained by hanging drop vapor diffusion in 1.6–1.8 M ammonium sulfate, 0.1 M Tris HCl pH 7.0–9.0. Crystals for P14/H-2D^b^/gp33, P14/H-2D^b^/V3P and P14/H-2D^b^/V3P_Y4F were obtained by hanging drop vapor diffusion in 19% PEG 6000, 0.1 M Tris HCl pH 8.0.

### Data collection, processing and structure determination

Data collection was performed at beam lines ID14-2 and ID23-2 at ESRF (Grenoble, France). Diffraction data were processed and scaled using MOSFLM 7.0.3 and SCALA [49]. Crystal structures were determined by molecular replacement using PHASER [50]. The crystal structure of H-2D^b^/gp33 (PDB ID: 1S7U) [2], with omitted peptide, was used as search model for H-2D^b^/V3P and H-2D^b^/V3P_Y4F. P14/H-2D^b^/gp33, P14/H-2D^b^/V3P and P14/H-2D^b^/V3P_Y4F were determined using 3PQY [51]. In all cases, poorer electron density was displayed for the TCR Cα domain, probably due to high flexibility, as previously observed [52]. Random 5% reflections were used for monitoring refinement by R_free_ cross-validation [53]. The model was rebuilt in Coot where necessary. The stereochemistry of the final models was verified using PROCHECK [54] or Coot [55].

### Circular dichroism (CD) analysis

Measurements were performed in 20mM K_2_HPO_4_/KH_2_PO_4_ (pH 7.5) using 0.15–0.3 mg/ml protein concentrations. Melting temperatures (Tm) were derived from changes in ellipticity at 218 nm as previously described [37]. Curves and Tm values are an average of at least three measurements from at least two independent refolding assays per pMHC. Spectra were analyzed using GraphPad Prism 5 (La Jolla, USA).

### Surface Plasmon Resonance (SPR) binding affinity analysis

All measurements were performed on BIAcore 2000 (GE Healthcare, USA) at 25°C. Soluble P14 (20 μg/ml) was non-covalently coupled to the anti-C_β_ antibody H57-597. 8000 RUs of H57-597 were coupled to a CM5-chip, resulting in 3000RUs immobilized P14. A control surface without antibody was used as reference. Concentration series of pMHCs were injected over the chip. The surface was regenerated with 40 μl 0.1 M Glycine-HCl, 500 mM NaCl, pH 2.5. Unspecific binding was corrected for by subtracting responses from reference flow cells. Data were analyzed with BIAevaluation 2000 (BIAcore AB, Uppsala, Sweden). K_D_-values were obtained from steady-state fitting of equilibrium binding curves from at least two independent measurements.

### Isothermal titration calorimetry (ITC)

Measurements were performed on a MicroCal iTC 200 (GE Healthcare, USA) at 25°C. 40 μl H-2D^b^/V3P (125μM) or H-2D^b^/gp33 (150 μM) in 10 mM Hepes, 150 mM NaCl, pH 7.4 were titrated into 300 μl of P14 (12.5–15 μM) in 10 injections under 1000 rpm stirring rate. Data analysis was performed using Origin, fitted to a non-linear curve in an iterative process. The reported constants are an average of two independent experiments.

### TCR down-regulation assays

P14-splenocytes were mixed with peptide-pulsed RMA cells at 10:1 effector:target (E:T) ratio. Cells were co-incubated at 37°C for 4 h and stained with anti-CD8β and -TCR Vα2 antibodies. Flow cytometry was performed using FACSCalibur (BD Biosciences) and changes in mean fluorescence intensity (MFI) of the Vα2 staining were used to estimate TCR down-regulation. Data was analyzed using Flowjo (Tree Star, Inc., Ashland, OR, USA).

### *In vivo* stimulation of P14 T cells and Cr^51^ release cytotoxicity assays

P14 TCR-transgenic mice were injected subcutaneously (SC) with 100 μg gp33 in PBS combined with 12.5 ng phosphorothioate-modified CpG-ODN 1668 (Invivogene, Sweden). 20 mg Aldara cream was applied at site of injection (5% imiquimod, Meda AB, Sweden). Animals were sacrificed 7 days later and spleens were recovered. Target RMA cells, labeled with Cr^51^, were pulsed with indicated peptide concentrations for 1 h at 37°C and subsequently mixed with *in vivo*-stimulated negatively selected (MACS CD8^+^ T cell isolation kit, Miltenyl Biotec, Germany) P14 CD8^+^ T cells at 3:1 E:T ratio followed by a standard 4h Cr^51^-release assay. Radioactivity was measured on a γ-counter (Wallac, Uppsala, Sweden). Percentage of specific lysis was calculated as [Cr^51^ release in test well–spontaneous Cr^51^ release] / [maximum Cr^51^ release–spontaneous Cr^51^ release] x 100.

### CD107a degranulation, intracellular IFNγ and TNF production

CD8^+^ T cells isolated from spleens of naïve (for TNF production assays) or *in vivo*-stimulated P14 transgenic mice were co-cultured for 5 h with 10^−6^ M or 10^−8^ M peptide-pulsed RMA cells in the presence of anti-CD107a antibody for degranulation assays. GolgiStop (BD Biosciences) was added after 1 h co-incubation. 4 h later, cells were stained with anti-CD8α and -CD3ԑ antibodies. For intracellular cytokine staining assays, cells were fixed and permeabilized using the Cytofix/Cytoperm kit (BD Biosciences) according to instructions. Cells were thereafter stained for IFNγ and TNF expression. FACS sampling was performed on CyAn (Dako, Glostrup, Denmark) and analyzed with FlowJo.

### MHC-I tetramer production

H-2D^b^ molecules with a biotinylation tag were refolded with peptides and mβ_2_m in the presence of protease inhibitors and purified as previously described [56]. Each obtained monomeric H-2D^b^/peptide complex (0.5 mg/ml) was tetramerized at a 4:1 ratio with streptavidin-PE or streptavidin-APC (BD Biosciences) in order to create each of the following tetramers H-2D^b^/gp33-(PE), H-2D^b^/Y4F-(APC), H-2D^b^/Y4F-(PE), H-2D^b^/V3P_Y4F-(APC) and H-2D^b^/V3P_Y4F-(PE).

### Identification of P14 CD8^+^ T cell responses upon LCMV vaccination

10^4^ P14 T cells (CD44^low^, Ly5.1^+^), isolated from spleens of P14 transgenic mice, were adoptively transferred intravenously (in PBS) into C57Bl/6 mice three days prior to intraperitoneal infection with 1x10^6^ PFU of LCMV (Clone 13). Spleens were harvested on day 7 post infection. CD8^+^ T cells were enriched by B cell panning and red blood cell lysis and stimulated with IL-2 (25 units/ml), Brefeldin A (5 μg/ml) (BD Biosciences) and 10^−6^ M peptide (gp33, Y4F or V3P_Y4F or no peptide) in complete RPMI for 5 h at 37°C, 5% CO_2_. Washed cells were surface stained with anti-CD8, -CD44 and -Ly5.1, fixed and permeabilized using BD cytofix/cytoperm kit (25 min at 4°C). Intracellular staining of IFNϒ, TNF and IL-2 (at 1:200) was performed for 30 min at 4°C. Endogenous T cells were distinguished by congenic marker Ly.5.2 from Ly.5.1^+^ P14 T cells. Cells were resuspended in FACS buffer after enrichment and stained at 1:400 for 1hr at RT with gp33, Y4F or V3P_Y4F tetramers. Washed cells were surface stained for CD8, Ly5.1, CD107a and CD44 for 30 min at 4°C. Data was collected using LSR Fortessa (BD Biosciences) and analyzed with Flowjo.

### Cloning of plasmids

pHW2000 vectors containing the 8 genes (PB2, PB1, PA, HA, NP, NA, M and NS), where NA and HA are derived from HKx31 (H3N2), and the internal genes from A/PR8/34 (PR8, H1N1), were constructed by reverse transcriptase-PCR (RT-PCR) amplification of the viral RNA. The peptides Y4F and V3P_Y4F were introduced into the Influenza A virus by inserting/replacing a region in the stalk of Neuraminidase (NA) using the cloning system as described.

### Viruses and cell culture

Reverse genetics, generation of modified Influenza: Briefly, 1 ug of each plasmid (NP, NS2, PB2, M, PA, PB1, HA and NA) was mixed with 16ug of lipofectomine and OptiMEM and added to a mix of co-cultured MDCK/293T cells, in the presence of TPCK-trypsin. The transfection was allowed to proceed for 48-72h in 5% CO_2_ at 37°C. The virus was thereafter propagated in chicken eggs for 2 days at 35° [57].

### RNA isolation and RT-PCR

Viral RNA was isolated from virus particles with RNeasy-Kit (Qiagen, Valencia, CA). Access RT-PCR kit (Promega) was used for characterization of recombinant influenza viruses.

### Identification of T cell responses upon influenza vaccination

Naive C57Bl6 mice were adoptively transferred with 10^4^ P14 T cells one day prior to infection. with 1 x10^4^ PFU or i.p. with 1.5 x10^7^ PFU of influenza A virus following anesthesia with isofluorane, then used for analysis of primary immunity at day 10 post infection. Kinetics, magnitude and phenotype of primary virus-specific CD8^+^ T cell responses were measured by flow cytometry. gp33- and APL-specific CD8^+^ T cell populations were characterized using H2D^b^/gp33, Y4F and V3P_Y4F tetramers. Splenocytes were incubated with tetramers for 60 min at room temperature. Washed cells were stained for CD8^+^ and CD44 for 30 min at 4°C. Intracellular IFNγ and TNF staining (1:200) was performed for 30 min at 4°C. Data was collected using LSR Fortessa (BD Biosciences) and analyzed with Flowjo.

### Statistical analysis

Data were routinely shown as mean ± SD. Unless stated otherwise, statistical significance was determined by the Student’s t test or analysis of variance (ANOVA) using GraphPad Prism 7.0. *P < 0.05; **P < 0.01; ***P < 0.001; ****P < 0.0001.

### Ethics statement

All experimental animal procedures were performed under Swedish national guidelines (N413/09) and following approval from the University of Melbourne animal ethics experimentation committee (ethics number 1312890.3).

## Supporting information

S1 Fig Isothermal calorimetry titration of P14 with H-2D^b^/gp33 (A) and H-2D^b^/V3P (B).
(TIF)

S2 Fig The p3P modification does not alter the conformation of the backbone of the APLs compared to their native counterparts. (TIF)

S3 Fig The p3P modification increases significantly P14 T cell responses. (TIF)

S4 Fig Vaccination of C57/Bl6 mice with influenza virus encoding for V3P_Y4F re-established efficient recognition of the immune escape variant Y4F in bronchoalveolar lavage (BAL) cells. (TIF)

S5 Fig Overall view of the ternary crystal structures of P14 in complex with H-2D^b^/gp33 (A), H-2D^b^/V3P (B) and H-2D^b^/V3P_Y4F (C).
(TIF)

S6 Fig Comparison of the crystal structures of the ternary complexes of P14/H-2Db/gp33 (A), P14/H-2Db/V3P (B) and P14/H-2Db/V3P_Y4F (C).
(TIF)

S7 Fig The T cell receptor P14 binds identically to H-2D^b^/gp33, H-2D^b^/V3P and H-2D^b^/V3P_Y4F. (TIF)

S8 Fig Simulated annealing omit maps were calculated for the structures of H-2D^b^/V3P, H-2D^b^/V3P_Y4F, P14/H-2D^b^/gp33, P14/H-2D^b^/V3P and P14/H-2D^b^/V3P_Y4F, respectively. (TIF)

S9 Fig The conformations of the H-2D^b^ residues R62, H155 and E163 are affected by the movements of the peptide residues p1K and p6F, following binding to the TCR P14. (TIF)

S10 Fig Conformation and contacts of H-2D^b^ hotspot residues, H155 (left) and R62 (right) in the three complexes, P14/H-2D^b^/gp33 (upper part), P14/H-2D^b^/V3P (middle) and P14/H-2D^b^/V3P_Y4F (lower part).
(TIF)

S1 Table Data collection and refinement statistics for H-2D^b^/V3P and H-2D^b^/V3P_Y4F. (DOCX)

S2 Table Data collection and refinement statistics of P14 in complex with H-2D^b^ presenting gp33, V3P or V3P_Y4F. (DOCX)

## References

[1] Yewdell JW, Bennink JR. Immunodominance in major histocompatibility complex class I-restricted T lymphocyte responses. Annu Rev Immunol. 1999;17:51–88. Epub 1999/06/08. 10.1146/annurev.immunol.17.1.51 . 10358753

[2] Velloso LM, Michaelsson J, Ljunggren HG, Schneider G, Achour A. Determination of structural principles underlying three different modes of lymphocytic choriomeningitis virus escape from CTL recognition. J Immunol. 2004;172(9):5504–11. Epub 2004/04/22. 10.4049/jimmunol.172.9.5504 . 15100292

[3] Achour A, Michaelsson J, Harris RA, Odeberg J, Grufman P, Sandberg JK, et al A structural basis for LCMV immune evasion: subversion of H-2D(b) and H-2K(b) presentation of gp33 revealed by comparative crystal structure.Analyses. Immunity. 2002;17(6):757–68. 10.1016/s1074-7613(02)00478-8 . 12479822

[4] Pircher H, Moskophidis D, Rohrer U, Burki K, Hengartner H, Zinkernagel RM. Viral escape by selection of cytotoxic T cell-resistant virus variants in vivo. Nature. 1990;346(6285):629–33. 10.1038/346629a0 . 1696684

[5] Petrovic D, Dempsey E, Doherty DG, Kelleher D, Long A. Hepatitis C virus—T-cell responses and viral escape mutations. European journal of immunology. 2012;42(1):17–26. Epub 2011/11/30. 10.1002/eji.201141593 . 22125159

[6] Gileadi U, Gallimore A, Van der Bruggen P, Cerundolo V. Effect of epitope flanking residues on the presentation of N-terminal cytotoxic T lymphocyte epitopes. European journal of immunology. 1999;29(7):2213–22. Epub 1999/07/31. 10.1002/(SICI)1521-4141(199907)29:07<2213::AID-IMMU2213>3.0.CO;2-8 [pii] . 10427984

[7] Seifert U, Liermann H, Racanelli V, Halenius A, Wiese M, Wedemeyer H, et al Hepatitis C virus mutation affects proteasomal epitope processing. The Journal of clinical investigation. 2004;114(2):250–9. Epub 2004/07/16. 10.1172/JCI20985 15254592 PMC449747

[8] Price GE, Ou R, Jiang H, Huang L, Moskophidis D. Viral escape by selection of cytotoxic T cell-resistant variants in influenza A virus pneumonia. The Journal of experimental medicine. 2000;191(11):1853–67. Epub 2000/06/06. 10.1084/jem.191.11.1853 10839802 PMC2213532

[9] Erickson AL, Kimura Y, Igarashi S, Eichelberger J, Houghton M, Sidney J, et al The outcome of hepatitis C virus infection is predicted by escape mutations in epitopes targeted by cytotoxic T lymphocytes. Immunity. 2001;15(6):883–95. Epub 2002/01/05. S1074-7613(01)00245-X [pii]. 10.1016/s1074-7613(01)00245-x . 11754811

[10] Butler NS, Theodossis A, Webb AI, Dunstone MA, Nastovska R, Ramarathinam SH, et al Structural and biological basis of CTL escape in coronavirus-infected mice. Journal of immunology. 2008;180(6):3926–37. Epub 2008/03/07. 180/6/3926 [pii]. 10.4049/jimmunol.180.6.3926 . 18322201

[11] Bertoletti A, Sette A, Chisari FV, Penna A, Levrero M, De Carli M, et al Natural variants of cytotoxic epitopes are T-cell receptor antagonists for antiviral cytotoxic T cells. Nature. 1994;369(6479):407–10. Epub 1994/06/02. 10.1038/369407a0 . 8196768

[12] Bowen DG, Walker CM. Mutational escape from CD8+ T cell immunity: HCV evolution, from chimpanzees to man. The Journal of experimental medicine. 2005;201(11):1709–14. Epub 2005/06/09. jem.20050808 [pii] 10.1084/jem.20050808 15939787 PMC2213256

[13] Goulder PJ, Watkins DI. HIV and SIV CTL escape: implications for vaccine design. Nat Rev Immunol. 2004;4(8):630–40. Epub 2004/08/03. 10.1038/nri1417 [pii]. . 15286729

[14] Barouch DH, Kunstman J, Kuroda MJ, Schmitz JE, Santra S, Peyerl FW, et al Eventual AIDS vaccine failure in a rhesus monkey by viral escape from cytotoxic T lymphocytes. Nature. 2002;415(6869):335–9. 10.1038/415335a . 11797012

[15] Valkenburg SA, Gras S, Guillonneau C, La Gruta NL, Thomas PG, Purcell AW, et al Protective efficacy of cross-reactive CD8+ T cells recognising mutant viral epitopes depends on peptide-MHC-I structural interactions and T cell activation threshold. PLoS Pathog. 2010;6(8). Epub 2010/08/17. 10.1371/journal.ppat.1001039 20711359 PMC2920842

[16] Purcell AW, McCluskey J, Rossjohn J. More than one reason to rethink the use of peptides in vaccine design. Nature reviews Drug discovery. 2007;6(5):404–14. 10.1038/nrd2224 . 17473845

[17] Chen W, Khilko S, Fecondo J, Margulies DH, McCluskey J. Determinant selection of major histocompatibility complex class I-restricted antigenic peptides is explained by class I-peptide affinity and is strongly influenced by nondominant anchor residues. The Journal of experimental medicine. 1994;180(4):1471–83. Epub 1994/10/01. 10.1084/jem.180.4.1471 7523572 PMC2191679

[18] Borbulevych OY, Baxter TK, Yu Z, Restifo NP, Baker BM. Increased immunogenicity of an anchor-modified tumor-associated antigen is due to the enhanced stability of the peptide/MHC complex: implications for vaccine design. Journal of immunology. 2005;174(8):4812–20. Epub 2005/04/09. 10.4049/jimmunol.174.8.4812 15814707 PMC2241749

[19] Pogue RR, Eron J, Frelinger JA, Matsui M. Amino-terminal alteration of the HLA-A*0201-restricted human immunodeficiency virus pol peptide increases complex stability and in vitro immunogenicity. Proc Natl Acad Sci U S A. 1995;92(18):8166–70. Epub 1995/08/29. 10.1073/pnas.92.18.8166 7545295 PMC41117

[20] Webb AI, Dunstone MA, Chen W, Aguilar MI, Chen Q, Jackson H, et al Functional and structural characteristics of NY-ESO-1-related HLA A2-restricted epitopes and the design of a novel immunogenic analogue. The Journal of biological chemistry. 2004;279(22):23438–46. Epub 2004/03/09. 10.1074/jbc.M314066200 . 15004033

[21] Kalergis AM, Ono T, Wang F, DiLorenzo TP, Honda S, Nathenson SG. Single amino acid replacements in an antigenic peptide are sufficient to alter the TCR V beta repertoire of the responding CD8+ cytotoxic lymphocyte population. Journal of immunology. 1999;162(12):7263–70. Epub 1999/06/08. . 10358174

[22] Butler NS, Theodossis A, Webb AI, Nastovska R, Ramarathinam SH, Dunstone MA, et al Prevention of cytotoxic T cell escape using a heteroclitic subdominant viral T cell determinant. PLoS Pathog. 2008;4(10):e1000186 Epub 2008/10/25. 10.1371/journal.ppat.1000186 18949029 PMC2563037

[23] van Stipdonk MJ, Badia-Martinez D, Sluijter M, Offringa R, van Hall T, Achour A. Design of agonistic altered peptides for the robust induction of CTL directed towards H-2Db in complex with the melanoma-associated epitope gp100. Cancer research. 2009;69(19):7784–92. 10.1158/0008-5472.CAN-09-1724 . 19789338

[24] Doorduijn EM, Sluijter M, Querido BJ, Oliveira CC, Achour A, Ossendorp F, et al TAP-independent self-peptides enhance T cell recognition of immune-escaped tumors. The Journal of clinical investigation. 2016;126(2):784–94. 10.1172/JCI83671 26784543 PMC4731191

[25] Hafstrand I, Doorduijn EM, Duru AD, Buratto J, Oliveira CC, Sandalova T, et al The MHC Class I Cancer-Associated Neoepitope Trh4 Linked with Impaired Peptide Processing Induces a Unique Noncanonical TCR Conformer. Journal of immunology. 2016;196(5):2327–34. 10.4049/jimmunol.1502249 . 26800871

[26] Hafstrand I, Doorduijn EM, Sun R, Talyzina A, Sluijter M, Pellegrino S, et al The Immunogenicity of a Proline-Substituted Altered Peptide Ligand toward the Cancer-Associated TEIPP Neoepitope Trh4 Is Unrelated to Complex Stability. Journal of immunology. 2018;200(8):2860–8. 10.4049/jimmunol.1700228 . 29507106

[27] Uchtenhagen H, Abualrous ET, Stahl E, Allerbring EB, Sluijter M, Zacharias M, et al Proline substitution independently enhances H-2D(b) complex stabilization and TCR recognition of melanoma-associated peptides. European journal of immunology. 2013;43(11):3051–60. 10.1002/eji.201343456 . 23939911

[28] Moskophidis D, Zinkernagel RM. Immunobiology of cytotoxic T-cell escape mutants of lymphocytic choriomeningitis virus. Journal of virology. 1995;69(4):2187–93. 7533851 10.1128/jvi.69.4.2187-2193.1995 PMC188887

[29] Aebischer T, Moskophidis D, Rohrer UH, Zinkernagel RM, Hengartner H. In vitro selection of lymphocytic choriomeningitis virus escape mutants by cytotoxic T lymphocytes. Proceedings of the National Academy of Sciences of the United States of America. 1991;88(24):11047–51. 10.1073/pnas.88.24.11047 1722316 PMC53070

[30] Puglielli MT, Zajac AJ, van der Most RG, Dzuris JL, Sette A, Altman JD, et al In vivo selection of a lymphocytic choriomeningitis virus variant that affects recognition of the GP33-43 epitope by H-2Db but not H-2Kb. Journal of virology. 2001;75(11):5099–107. 10.1128/JVI.75.11.5099-5107.2001 11333891 PMC114915

[31] Jenkins MR, Webby R, Doherty PC, Turner SJ. Addition of a prominent epitope affects influenza A virus-specific CD8+ T cell immunodominance hierarchies when antigen is limiting. Journal of immunology. 2006;177(5):2917–25. 10.4049/jimmunol.177.5.2917 . 16920927

[32] Martin S, Kohler H, Weltzien HU, Leipner C. Selective activation of CD8 T cell effector functions by epitope variants of lymphocytic choriomeningitis virus glycoprotein. Journal of immunology. 1996;157(6):2358–65. . 8805633

[33] Tian S, Maile R, Collins EJ, Frelinger JA. CD8+ T cell activation is governed by TCR-peptide/MHC affinity, not dissociation rate. Journal of immunology. 2007;179(5):2952–60. Epub 2007/08/22. 179/5/2952 [pii]. 10.4049/jimmunol.179.5.2952 . 17709510

[34] Tissot AC, Ciatto C, Mittl PR, Grutter MG, Pluckthun A. Viral escape at the molecular level explained by quantitative T-cell receptor/peptide/MHC interactions and the crystal structure of a peptide/MHC complex. Journal of molecular biology. 2000;302(4):873–85. Epub 2000/09/20. 10.1006/jmbi.2000.4501 [pii]. . 10993729

[35] Dyall R, Bowne WB, Weber LW, LeMaoult J, Szabo P, Moroi Y, et al Heteroclitic immunization induces tumor immunity. The Journal of experimental medicine. 1998;188(9):1553–61. 10.1084/jem.188.9.1553 9802967 PMC2212523

[36] Trujillo JA, Gras S, Twist KA, Croft NP, Channappanavar R, Rossjohn J, et al Structural and functional correlates of enhanced antiviral immunity generated by heteroclitic CD8 T cell epitopes. Journal of immunology. 2014;192(11):5245–56. 10.4049/jimmunol.1400111 24795457 PMC4052115

[37] Allerbring EB, Duru AD, Uchtenhagen H, Madhurantakam C, Tomek MB, Grimm S, et al Unexpected T-cell recognition of an altered peptide ligand is driven by reversed thermodynamics. European journal of immunology. 2012;42(11):2990–3000. 10.1002/eji.201242588 . 22837158

[38] Bhattacharyya R, Chakrabarti P. Stereospecific interactions of proline residues in protein structures and complexes. Journal of molecular biology. 2003;331(4):925–40. Epub 2003/08/12. S0022283603007599 [pii]. 10.1016/s0022-2836(03)00759-9 . 12909019

[39] Biedermannova L, K ER, Berka K, Hobza P, Vondrasek J. Another role of proline: stabilization interactions in proteins and protein complexes concerning proline and tryptophane. Phys Chem Chem Phys. 2008;10(42):6350–9. Epub 2008/10/31. 10.1039/b805087b . 18972023

[40] Yu H, Zhao Y, Guo C, Gan Y, Huang H. The role of proline substitutions within flexible regions on thermostability of luciferase. Biochim Biophys Acta. 2015;1854(1):65–72. Epub 2014/12/03. 10.1016/j.bbapap.2014.10.017 [pii]. . 25448017

[41] Haidar JN, Zhu W, Lypowy J, Pierce BG, Bari A, Persaud K, et al Backbone flexibility of CDR3 and immune recognition of antigens. Journal of molecular biology. 2014;426(7):1583–99. Epub 2014/01/02. 10.1016/j.jmb.2013.12.024 [pii]. . 24380763

[42] Ayres CM, Corcelli SA, Baker BM. Peptide and Peptide-Dependent Motions in MHC Proteins: Immunological Implications and Biophysical Underpinnings. Front Immunol. 2017;8:935 Epub 2017/08/22. 10.3389/fimmu.2017.00935 28824655 PMC5545744

[43] Garboczi DN, Ghosh P, Utz U, Fan QR, Biddison WE, Wiley DC. Structure of the complex between human T-cell receptor, viral peptide and HLA-A2. Nature. 1996;384(6605):134–41. 10.1038/384134a0 . 8906788

[44] Armstrong KM, Insaidoo FK, Baker BM. Thermodynamics of T-cell receptor-peptide/MHC interactions: progress and opportunities. Journal of molecular recognition: JMR. 2008;21(4):275–87. 10.1002/jmr.896 18496839 PMC3674762

[45] Wang B, Sharma A, Maile R, Saad M, Collins EJ, Frelinger JA. Peptidic termini play a significant role in TCR recognition. J Immunol. 2002;169(6):3137–45. 10.4049/jimmunol.169.6.3137 . 12218131

[46] Baker BM, Turner RV, Gagnon SJ, Wiley DC, Biddison WE. Identification of a crucial energetic footprint on the alpha1 helix of human histocompatibility leukocyte antigen (HLA)-A2 that provides functional interactions for recognition by tax peptide/HLA-A2-specific T cell receptors. The Journal of experimental medicine. 2001;193(5):551–62. 10.1084/jem.193.5.551 11238586 PMC2193388

[47] Baxter TK, Gagnon SJ, Davis-Harrison RL, Beck JC, Binz AK, Turner RV, et al Strategic mutations in the class I major histocompatibility complex HLA-A2 independently affect both peptide binding and T cell receptor recognition. The Journal of biological chemistry. 2004;279(28):29175–84. 10.1074/jbc.M403372200 . 15131131

[48] Achour A, Harris RA, Persson K, Sundback J, Sentman CL, Schneider G, et al Murine class I major histocompatibility complex H-2Dd: expression, refolding and crystallization. Acta Crystallogr D Biol Crystallogr. 1999;55(Pt 1):260–2. Epub 1999/03/25. 10.1107/S0907444998005265 [pii]. . 10089418

[49] Evans P. Scaling and assessment of data quality. Acta crystallographica Section D, Biological crystallography. 2006;62(Pt 1):72–82. Epub 2005/12/22. 10.1107/S0907444905036693 . 16369096

[50] McCoy AJ, Grosse-Kunstleve RW, Adams PD, Winn MD, Storoni LC, Read RJ. Phaser crystallographic software. J Appl Crystallogr. 2007;40(Pt 4):658–74. Epub 2007/08/01. 10.1107/S0021889807021206 19461840 PMC2483472

[51] Day EB, Guillonneau C, Gras S, La Gruta NL, Vignali DA, Doherty PC, et al Structural basis for enabling T-cell receptor diversity within biased virus-specific CD8+ T-cell responses. Proceedings of the National Academy of Sciences of the United States of America. 2011;108(23):9536–41. Epub 2011/05/25. 1106851108 [pii] 10.1073/pnas.1106851108 21606376 PMC3111262

[52] Dunn SM, Rizkallah PJ, Baston E, Mahon T, Cameron B, Moysey R, et al Directed evolution of human T cell receptor CDR2 residues by phage display dramatically enhances affinity for cognate peptide-MHC without increasing apparent cross-reactivity. Protein Sci. 2006;15(4):710–21. Epub 2006/04/08. 15/4/710 [pii] 10.1110/ps.051936406 16600963 PMC2242494

[53] Brunger AT. Free R value: a novel statistical quantity for assessing the accuracy of crystal structures. Nature. 1992;355(6359):472–5. Epub 1992/01/30. 10.1038/355472a0 . 18481394

[54] Laskowski RA, Moss DS, Thornton JM. Main-chain bond lengths and bond angles in protein structures. Journal of molecular biology. 1993;231(4):1049–67. 10.1006/jmbi.1993.1351 . 8515464

[55] Emsley P, Cowtan K. Coot: model-building tools for molecular graphics. Acta Crystallogr D Biol Crystallogr. 2004;60(Pt 12 Pt 1):2126–32. Epub 2004/12/02. S0907444904019158 [pii] 10.1107/S0907444904019158 . 15572765

[56] Michaelsson J, Achour A, Salcedo M, Kase-Sjostrom A, Sundback J, Harris RA, et al Visualization of inhibitory Ly49 receptor specificity with soluble major histocompatibility complex class I tetramers. European journal of immunology. 2000;30(1):300–7. Epub 1999/12/22. 10.1002/1521-4141(200001)30:1<300::AID-IMMU300>3.0.CO;2-S . 10602053

[57] Hoffmann E, Neumann G, Kawaoka Y, Hobom G, Webster RG. A DNA transfection system for generation of influenza A virus from eight plasmids. Proceedings of the National Academy of Sciences of the United States of America. 2000;97(11):6108–13. 10.1073/pnas.100133697 10801978 PMC18566

