# Supplementary figures and images for: Tuning antiviral CD8 T-cell response via proline-altered peptide ligand vaccination
Source: PLoS Pathog. 2020 May 4;16(5):e1008244. doi: 10.1371/journal.ppat.1008244 (PMC7224568; doi:10.1371/journal.ppat.1008244)

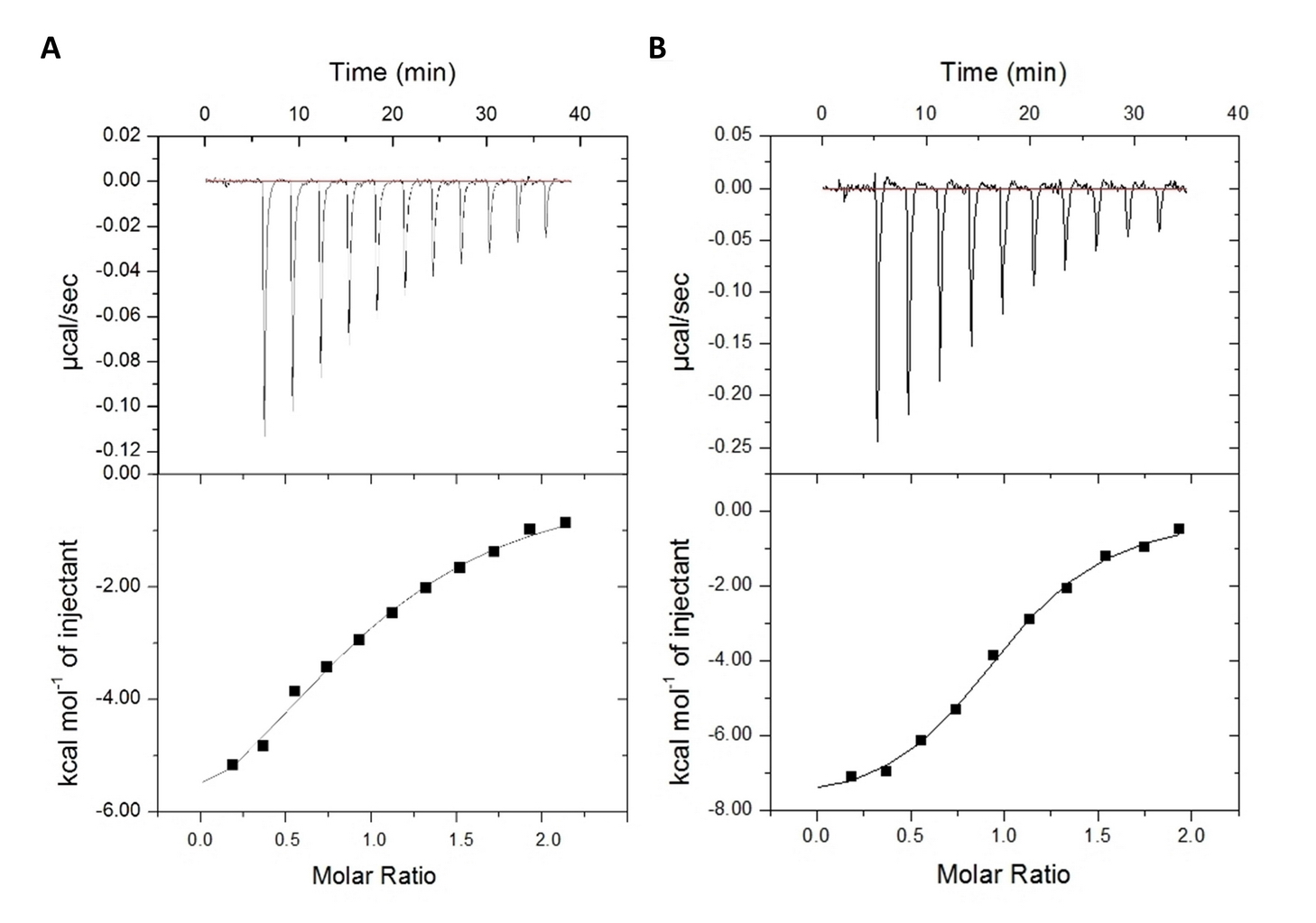

Supplement: S1 Fig — Isothermal calorimetry titration of P14 with H-2Db/gp33 (A) and H-2Db/V3P (B). (TIF) [file ppat.1008244.s001.tif]

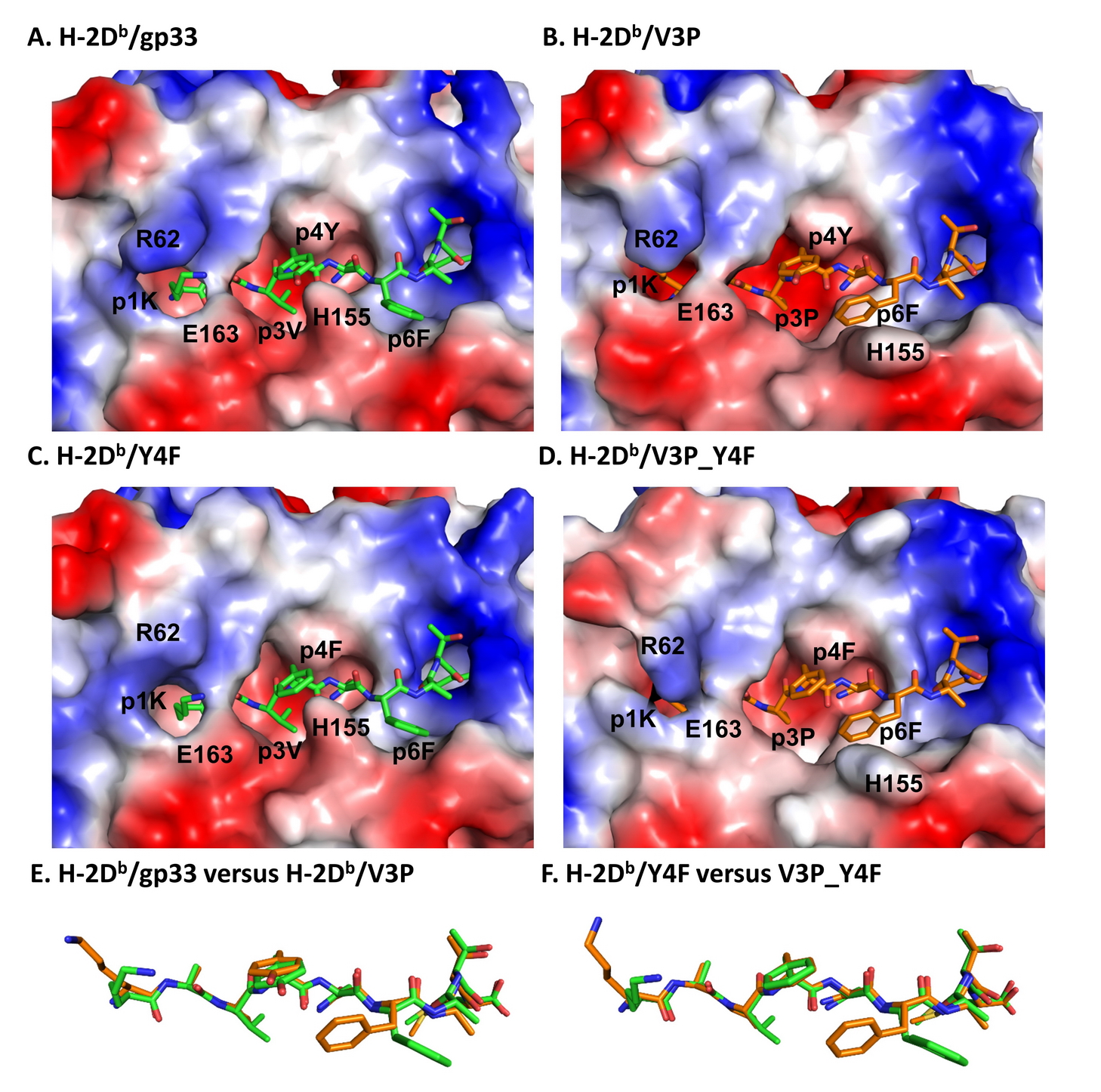

Supplement: S2 Fig — (TIF) [file ppat.1008244.s002.tif]

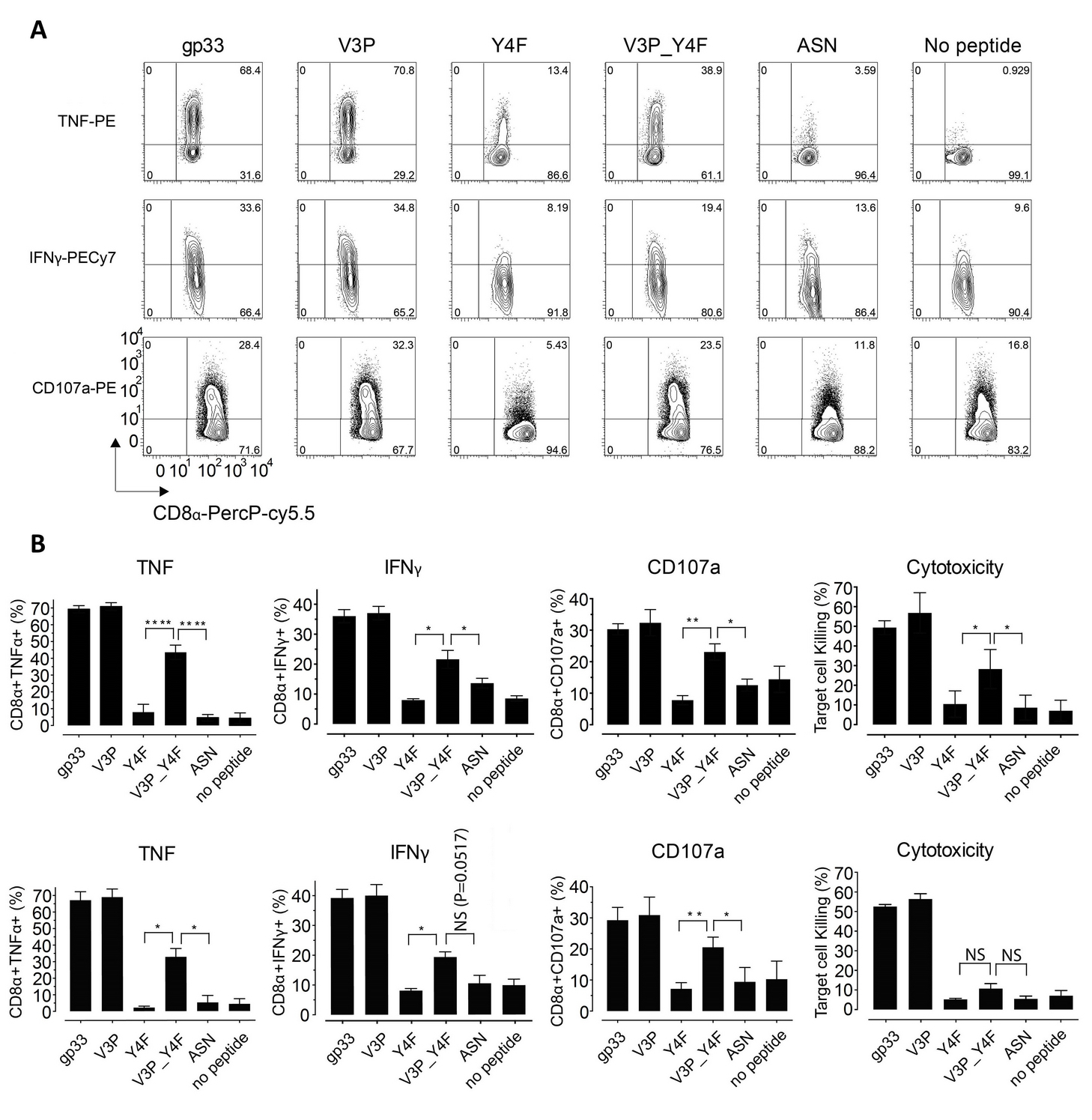

Supplement: S3 Fig — (TIF) [file ppat.1008244.s003.tif]

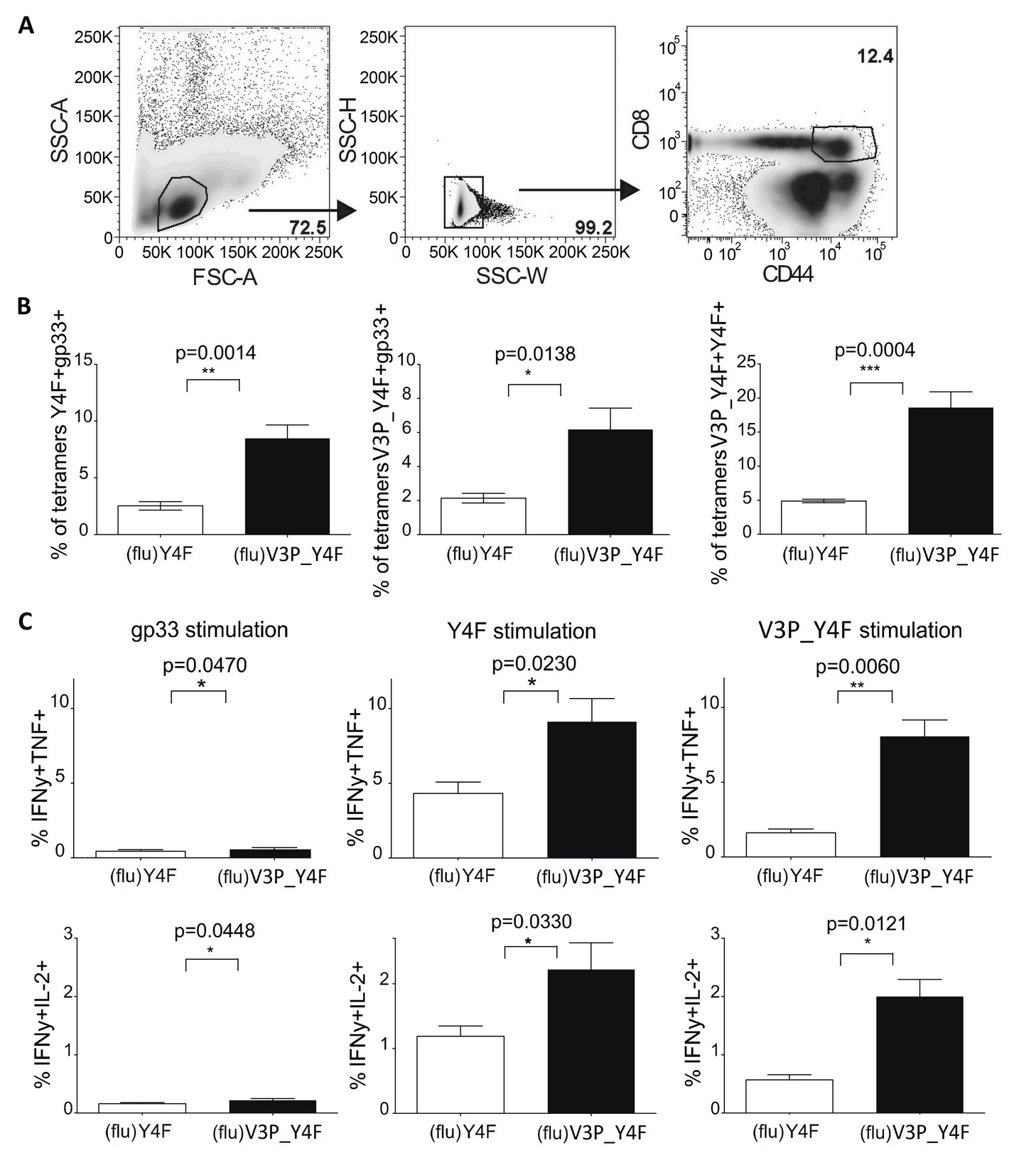

Supplement: S4 Fig — (TIF) [file ppat.1008244.s004.tif]

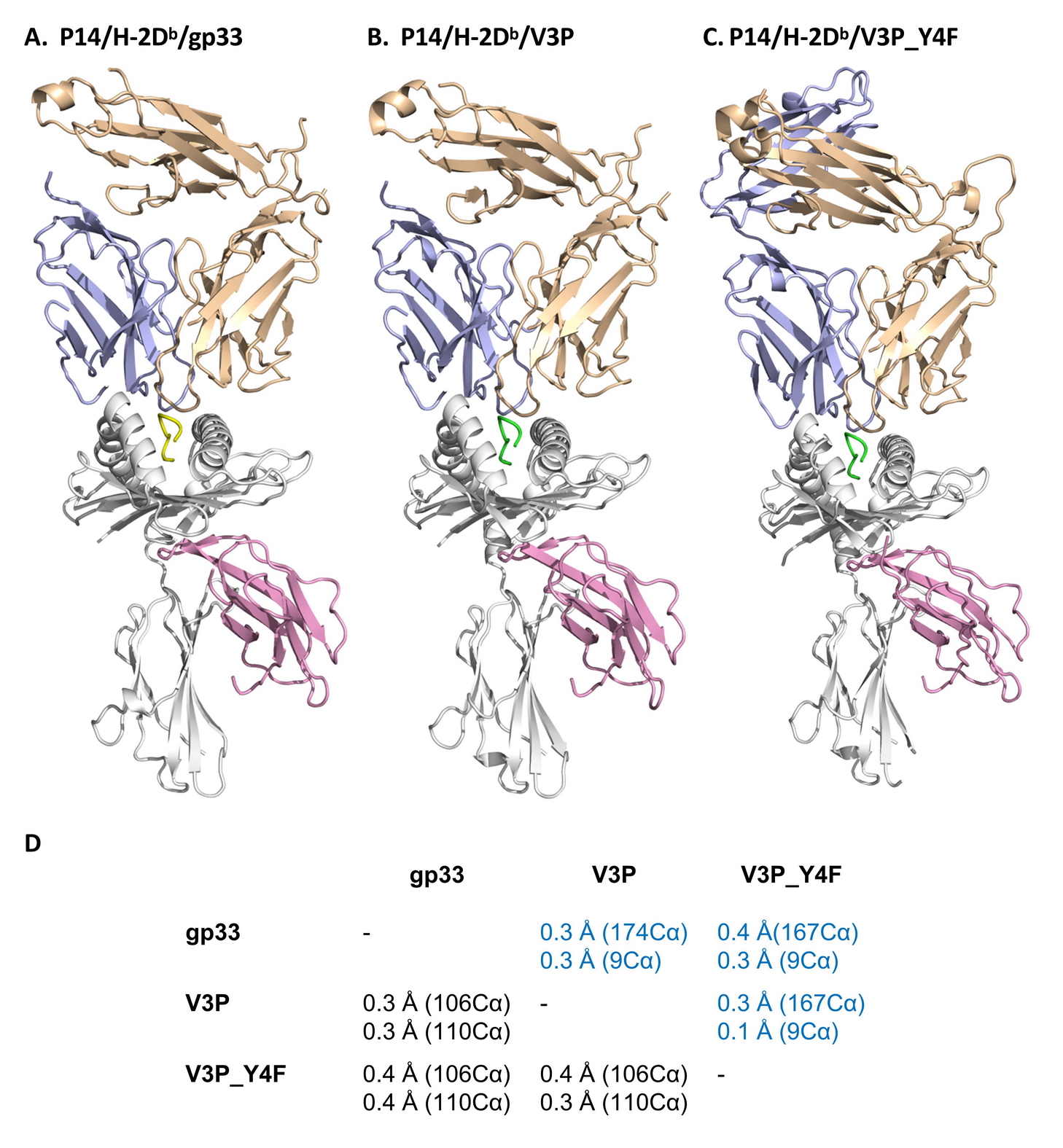

Supplement: S5 Fig — Overall view of the ternary crystal structures of P14 in complex with H-2Db/gp33 (A), H-2Db/V3P (B) and H-2Db/V3P_Y4F (C). (TIF) [file ppat.1008244.s005.tif]

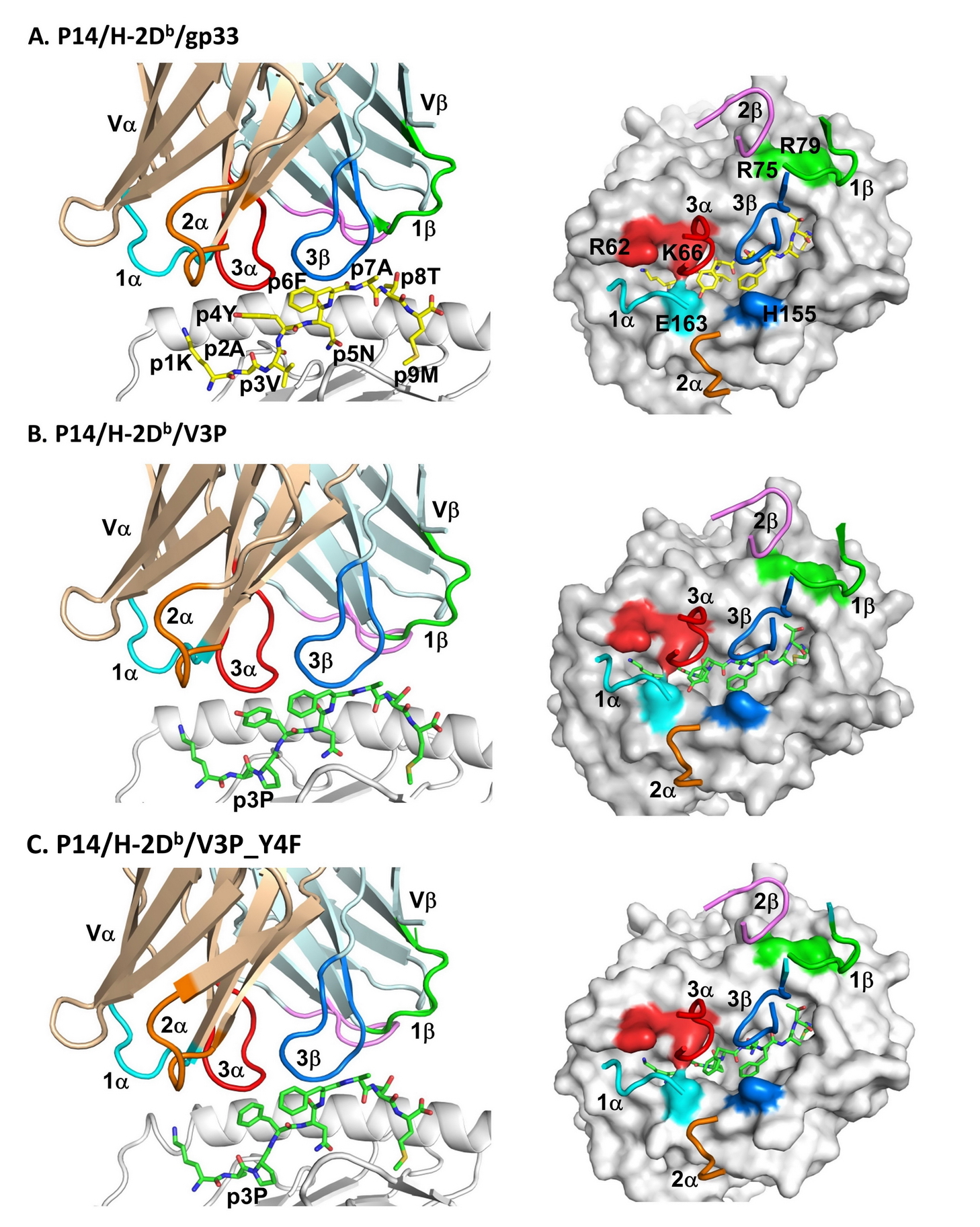

Supplement: S6 Fig — Comparison of the crystal structures of the ternary complexes of P14/H-2Db/gp33 (A), P14/H-2Db/V3P (B) and P14/H-2Db/V3P_Y4F (C). (TIF) [file ppat.1008244.s006.tif]

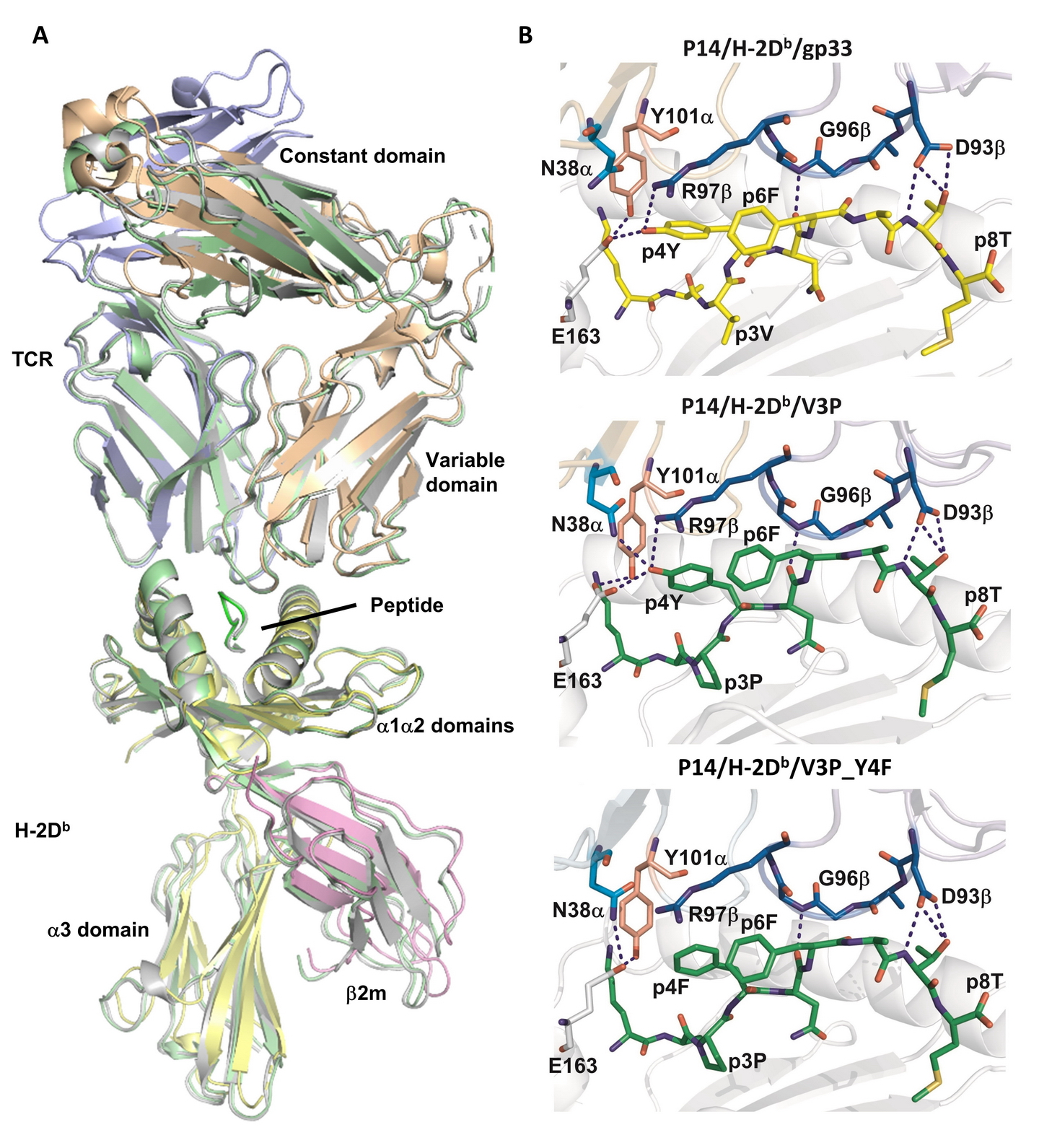

Supplement: S7 Fig — (TIF) [file ppat.1008244.s007.tif]

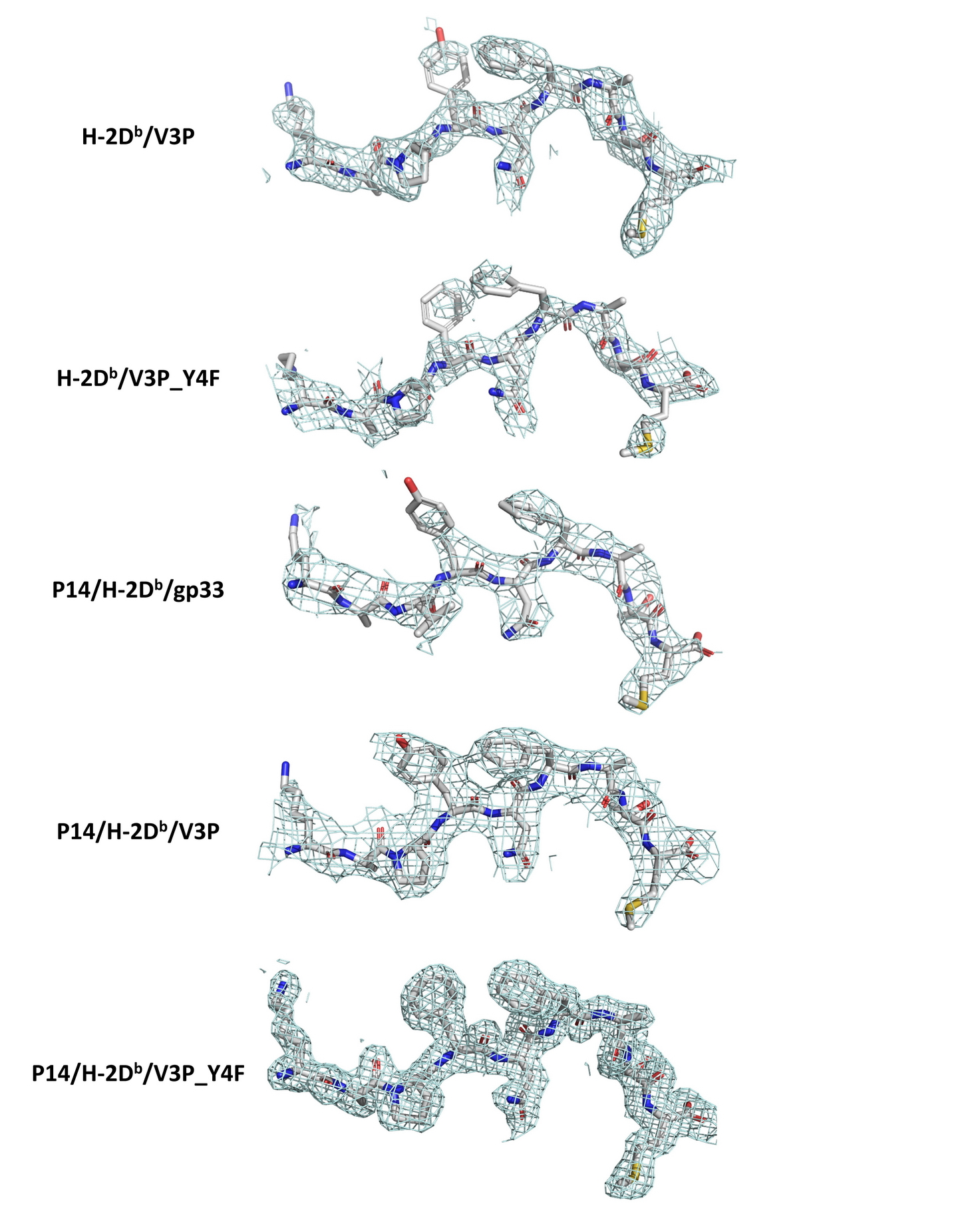

Supplement: S8 Fig — (TIF) [file ppat.1008244.s008.tif]

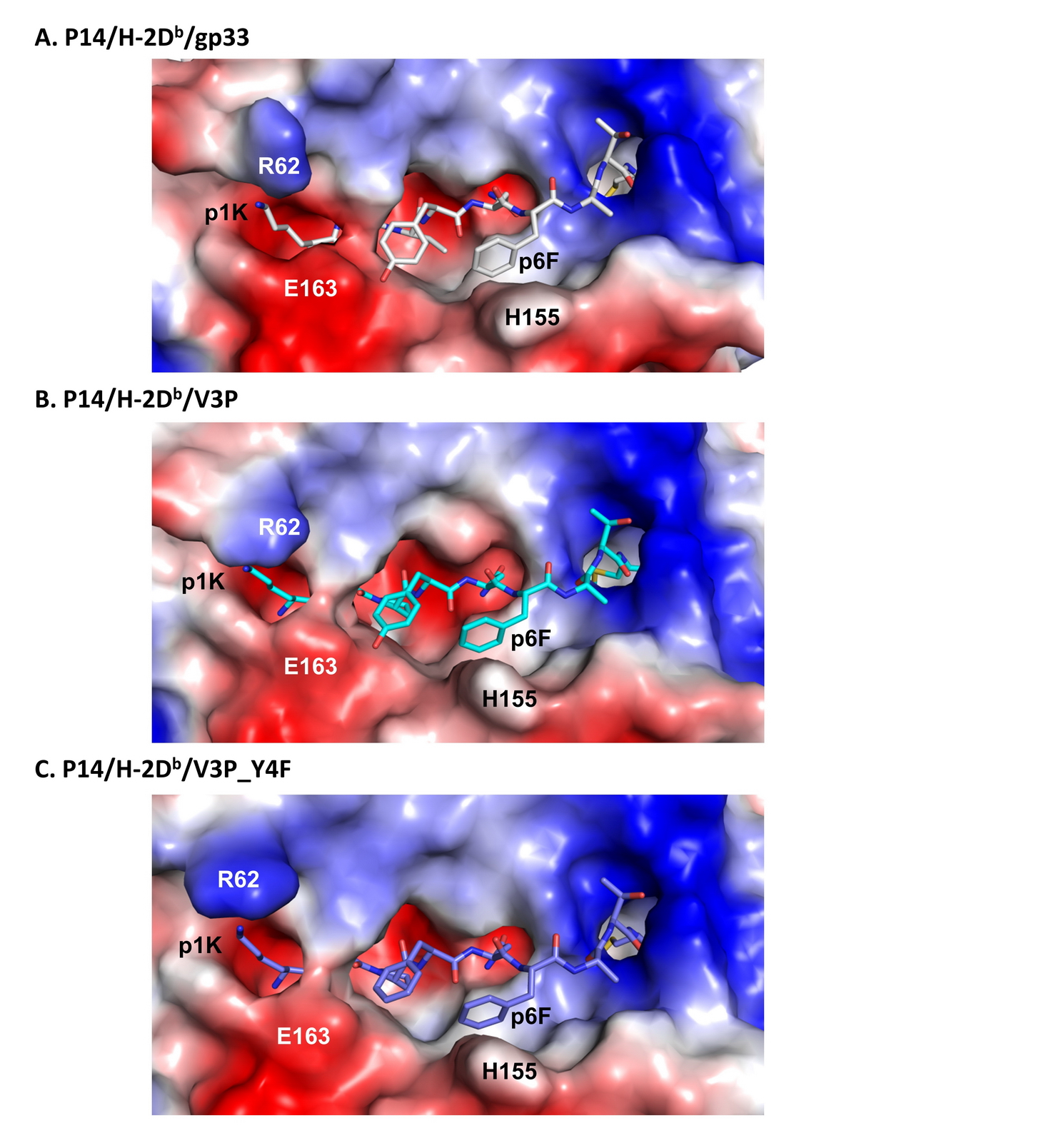

Supplement: S9 Fig — (TIF) [file ppat.1008244.s009.tif]

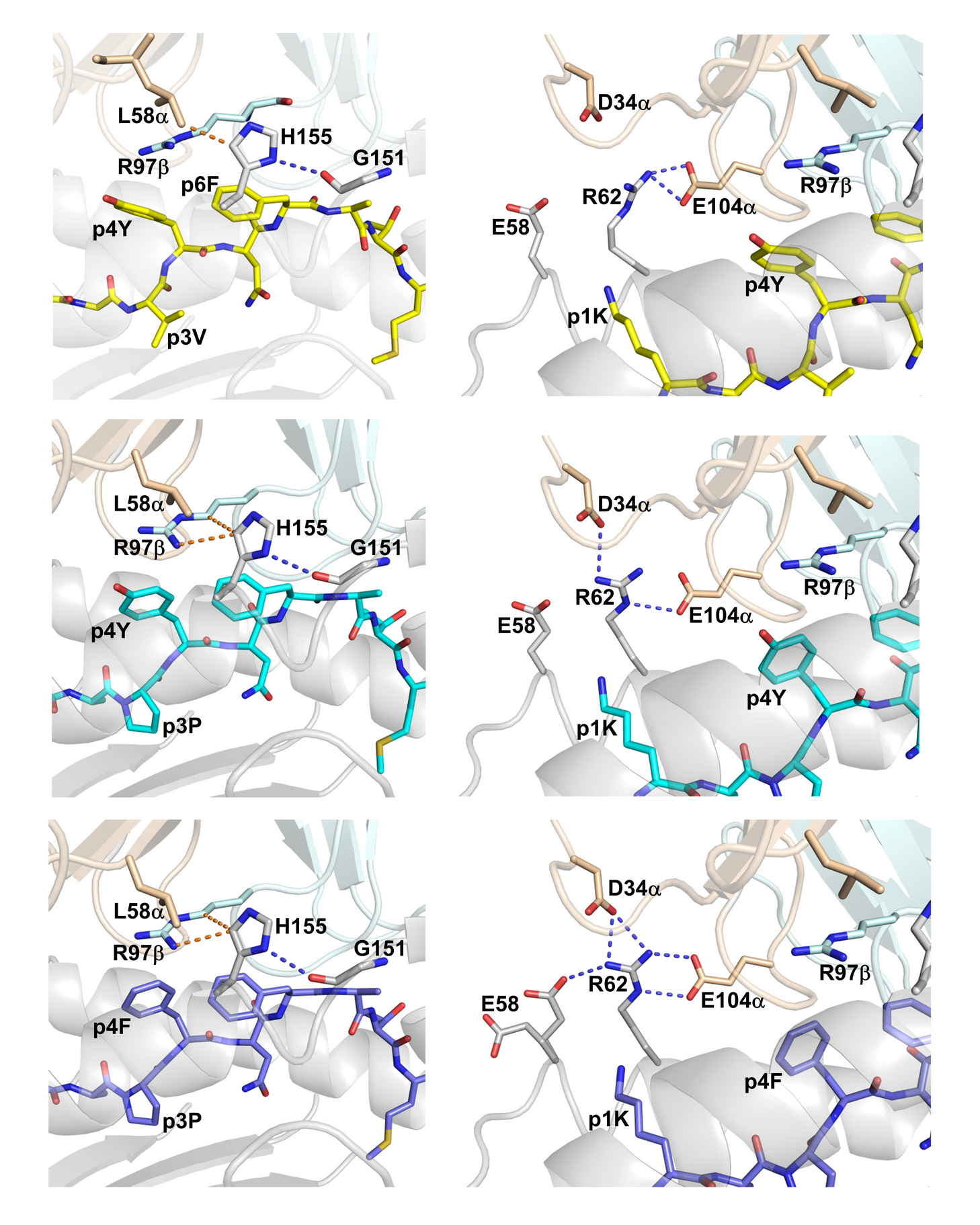

Supplement: S10 Fig — Conformation and contacts of H-2Db hotspot residues, H155 (left) and R62 (right) in the three complexes, P14/H-2Db/gp33 (upper part), P14/H-2Db/V3P (middle) and P14/H-2Db/V3P_Y4F (lower part). (TIF) [file ppat.1008244.s010.tif]
