# Supplementary material for: Tuning antiviral CD8 T-cell response via proline-altered peptide ligand vaccination
Source: PLoS Pathog. 2020 May 4;16(5):e1008244. doi: 10.1371/journal.ppat.1008244 (PMC7224568; doi:10.1371/journal.ppat.1008244)
Supplement: S1 Table — (DOCX) [file ppat.1008244.s011.docx]

**Supplementary Table I. Data collection and refinement statistics for H-2D^b^/V3P and H-2D^b^/V3P_Y4F**

| Name | H-2D^b^/V3P /KAPYNFATM | H-2D^b^/V3P_Y4F /KAPFNFATM |
| --- | --- | --- |
| PDB | 4NSK | 3TBY |
| Space group | C2 | P2_1_ |
| Cell dimensions |  |  |
| *a* (Å) | 90.5 | 92.8 |
| *b* (Å) | 108.5 | 124.3 |
| *c* (Å) | 57.4 | 99.6 |
| β (^o^) | 122.4 | 103.3 |
| Resolution | 54.9-2.6 (2.71-2.57) | 48.5-2.5 (2.65-2.50) |
| N_unique_ | 14534 (1986) | 75693 (11100) |
| Multiplicity | 2.4 (2.2) | 3.7 (3.6) |
| Completeness (%) | 94.0 (88.2) | 98.1 (99.2) |
| I/σ(I) | 6.9 (1.7) | 10.7 (1.9) |
| R_merge_ | 0.100 (0.553) | 0.112 (0.631) |
| Complexes in au | 1 | 4 |
| Refinement |  |  |
| R _cryst_ (%) | 21.6 | 28.5 |
| R _free_ (%) | 28.3 | 31.5 |
| Number of protein atoms/residues | 3142 / 384 | 12308 / 1532 |
| Number of water molecules | 45 | 232 |
|  |  |  |
| Rmsd from ideal geometry |  |  |
| Bond length (Å) | 0.011 | 0.010 |
| Bond angles (^o^) | 1.32 | 1.19 |
| Ramachandran plot |  |  |
| Residues in preferred regions (%) | 92.25 | 94.16 |
| Outliers (%) | 0.53 | 0.63 |
| Mean B-values (Å2) |  |  |
| <B> for MHC | 49.1 | 53.9 |
| <B> for Peptide | 45.6 | 64.8 |
| <B> for waters | 36.9 | 48.2 |

R_merge_=Σ|I_i_−I_m_| / Σ I_i_, where Ii is the intensity of the measured reflection, and I_m_ is the mean intensity for all observations of that reflection. Numbers within parentheses are for the outer resolution shell of data.
