# Supplementary material for: Tuning antiviral CD8 T-cell response via proline-altered peptide ligand vaccination
Source: PLoS Pathog. 2020 May 4;16(5):e1008244. doi: 10.1371/journal.ppat.1008244 (PMC7224568; doi:10.1371/journal.ppat.1008244)
Supplement: S2 Table — (DOCX) [file ppat.1008244.s012.docx]

**Supplementary Table II. Data collection and refinement statistics of P14 in complex with H-2D^b^ presenting gp33, V3P or V3P_Y4F**

|  | gp33  KAVYNFATM | V3P  KAPYNFATM | V3P_Y4F KAPFNFATM |
| --- | --- | --- | --- |
| PDB | 5TJE | 5TIL | 5M02 |
| Space group | P2_1_2_1_2_1_ | P2_1_2_1_2_1_ | C2 |
| Cell dimensions |  |  |  |
| *a* (Å) | 61.17 | 61.22 | 255.11 |
| *b* (Å) | 66.94 | 66.70 | 46.50 |
| *c* (Å) | 525.39 | 523.48 | 89.02 |
| β (^o^) | 90 | 90 | 94.6 |
| Resolution | 38.88-3.2  (3.37-3.2) | 47.38-2.83  (2.98-2.83) | 127-1.75  (1.81-1.75) |
| N_unique_ | 35190 (5166) | 51497 (19359) | 103816 (5042) |
| Multiplicity | 3.2 (3.2) | 3.0 (2.8) | 3.7 (3.7) |
| Completeness (%) | 96.1 (98.1) | 97.8 (90.8) | 98.5 (96.3) |
| I/σ(I) | 5.8 (1.7) | 6.1 (1.7) | 13.5 (1.4) |
| R_merge_ | 0.170 (0.715) | 0.125 (0.561) | 0.043 (0.77) |
| N complexes in au | 2 | 2 | 1 |
| Refinement |  |  |  |
| R cryst (%) | 22.8 | 22.32 | 18.79 |
| R free (%) | 31.3 | 27.35 | 21.38 |
| Number of protein atoms/residues | 11757/1467 | 11811/ 1470 | 6587/ |
| Number of water molecules | 27 | 40 | 392 |
|  |  |  |  |
| Rmsd from ideal geometry |  |  |  |
| Bond length (Å) | 0.014 | 0.010 | 0.011 |
| Bond angles (^o^) | 1.7 | 1.43 | 1.52 |
| Ramachandran plot |  |  |  |
| Residues in preferred regions (%) | 91 | 92 | 96.7 |
| Outliers (%) | 0.6 | 0.3 | 0.38 |
| Mean B-values (Å2) | 79 | 72 | 22 |
| <B> for MHC A/B | 78/68 | 72/61 | 17/21 |
| <B> for Peptide | 56 | 49 | 22 |
| <B> for TCR A/B | 74/88 | 76/69 | 29/20 |
| <B> for waters | 36 | 54 | 39 |

R_merge_=Σ|I_i_−I_m_| / Σ I_i_, where Ii is the intensity of the measured reflection, and I_m_ is the mean intensity for all observations of that reflection. Numbers within parentheses are for the outer resolution shell of data.
